# Head and Neck Paraganglioma (HNPGL) Registry: A study protocol for prospective data collection in patients with Head and Neck Paragangliomas

**DOI:** 10.1371/journal.pone.0307311

**Published:** 2024-07-25

**Authors:** Carolijn J. M. de Bresser, Bernadette P. M. van Nesselrooij, Mark J. C. van Treijen, Arthur J. A. T. Braat, Mischa de Ridder, Robert J. Stokroos, Remco de Bree, Gert J. de Borst, Johannes A. Rijken, Bart-Jeroen Petri

**Affiliations:** 1 Department of Vascular Surgery, University Medical Center Utrecht, Utrecht, The Netherlands; 2 Department of Clinical Genetics, University Medical Center Utrecht, Utrecht, The Netherlands; 3 Department of Endocrine Oncology, University Medical Center Utrecht, Utrecht, The Netherlands; 4 Department of Radiology and Nuclear Medicine, University Medical Center Utrecht, Utrecht, The Netherlands; 5 Department of Nuclear Medicine, Netherlands Cancer Institute, Amsterdam, The Netherlands; 6 Department of Radiotherapy, University Medical Center Utrecht, Utrecht, The Netherlands; 7 Department of Otolaryngology-Head and Neck Surgery, University Medical Center Utrecht, Utrecht, The Netherlands; 8 Department of Head and Neck Surgical Oncology, University Medical Center Utrecht, Utrecht, The Netherlands; 2nd Medical Faculty Charles University Prague and Faculty Hospital Motol, CZECHIA

## Abstract

**Introduction:**

There is a lack of comprehensive and uniform data on head and neck paragangliomas (HNPGLs), and research is challenging due to its rarity and the involvement of multiple medical specialties. To improve current research data collection, we initiated the Head and Neck Paraganglioma Registry (HNPGL Registry). The aim of the HNPGL Registry is to a) collect extensive data on all HNPGL patients through a predefined protocol, b) give insight in the long term outcomes using patient reported outcome measures (PROMs), c) create uniformity in the diagnostic and clinical management of these conditions, and thereby d) help provide content for future (randomized) research.

**Methods and analysis:**

The HNPGL Registry is designed as a prospective longitudinal observational registry for data collection on HNPGL patients and carriers of (likely) pathogenic variants causative of HNPGLs. All patients, regardless of the received treatment modality, can be included in the registry after informed consent is obtained. All relevant data regarding the initial presentation, diagnostics, treatment, and follow-up will be collected prospectively in an electronic case report form. In addition a survey containing the EuroQol 5D-5L (EQ-5D-5L), European Organisation for Research and Treatment of Cancer Quality of Life Questionnaire (EORTC QLQ-C30), Modified Fatigue Impact Scale (MFIS), Short QUestionnaire to Assess Health-enhancing physical activity (SQUASH), Cancer Worry Scale (CWS) and Hospital Anxiety and Depression Scale (HADS) will be sent periodically. The registry protocol was approved by the Medical Ethical Review Board of the University Medical Center Utrecht.

**Conclusion:**

The HNPGL Registry data will be used to further establish the optimal management for HNPGL patients and lay the foundation for guideline recommendations and the outline of future research.

## Introduction

### Evidence gap

Head and neck paragangliomas (HNPGLs) are rare tumors, comprising only 0.6% of head and neck tumors and 0.03% of all tumors, with an overall incidence of 1 in 30,000 to 1 in 1,000,000 [1–3]. The most common types of HNPGLs are the carotid body tumors or carotid body paragangliomas (CBPGL), jugular paragangliomas (JPGL), vagal paragangliomas (VPGL), and tympanic paragangliomas (TPGLs) [4]. Computed tomography (CT) and magnetic resonance imaging (MRI) are modalities often used for diagnostic anatomical imaging, whereas diagnostic functional imaging with iodine-123 metaiodobenzylguanidine (MIBG), fluor-18-fludeoxyglucose (FDG), fluoro-18-dihydroxyphenylalanine (FDOPA) or gallium-68-dotaphenyltyrosineoctreotide (DOTATOC) are used for discrimination between paragangliomas and other lesions [5–8]. Treatment options for different types of HNPGLs vary, including wait-and-scan, surgical resection, radiotherapy, embolization or a combination of these. Targeted radionuclide therapies using iodine-131-metaiodobenzylguanidine (targeting the norepinephrine transporters) and peptide receptor radionuclide therapy (somatostatin analogues radiolabeled with either Lutetium-177 or Yttrium-90) have been presented as promising therapeutic options in the management of metastatic or inoperable paragangliomas (PGLs) [9]. Recent development has shown further improvements of these radiopharmaceuticals by exchanging the beta-emitting isotopes with alpha-emitting isotopes (e.g. Astatine-211 and Actinium-225). These radiopharmaceuticals are currently investigated in clinical trials (JRCT ID: jRCT2021220012 and Clinicaltrials.gov identifier NCT05477576) and results are eagerly awaited.

PGLs are often caused by (likely) pathogenic variants in the succinate dehydrogenase complex (SDHx), with different germline variants leading to different PGL syndromes. Over 40% of all PGL syndromes is caused by germline variants [10, 11]. Increasing insight has been obtained of the different SDHx syndromes and their clinical heterogeneity with marked differences in tumor multifocality, growth of associated tumors, malignant behavior, and mortality rates [10, 12]. The expanding landscape of recent new genes identified for potentially predisposing people to the development of HNPGLs, opens up a new field of possible different characteristic biological behaviors [13].

Despite a better understanding of the disease, many questions remain unanswered. The debate on the frequency and most optimal imaging modality is far from closed. The effect and impact of active surveillance on mortality, morbidity, and quality of life for example, is still ambiguous and no consensus has been obtained on the ideal follow-up regimen. The disadvantages of surgery or radiotherapy often outweigh the limited risks that can arise in the natural course of HNPGLs, leading to a shift towards a wait-and-scan approach [3, 14]. Surgical resection of HNPGLs may be complicated by (permanent or temporary) cranial nerve injury, blood loss and even death or stroke in severe cases [15, 16]. Radiotherapy obtains local tumor control in 88–100% of HNPGL cases, but may cause radiation-induced sarcoma with an incidence of 0.06–0.17% in the head-and-neck area and a relatively poor 5-year overall-free survival rate ranging from 32–58% [17–19].

Considering the many knowledge gaps around the diagnostic and intervention process of HNPGLs, patients benefit from high quality studies. Therefore, the HNPGL Registry aims to a) collect comprehensive data on all HNPGL patients using a standardized protocol, b) provide insights into long-term outcomes using patient reported outcome measures (PROMs), c) create uniformity in the diagnosis and clinical management of these conditions, and thereby d) contribute to future (randomized) research endeavors.

The HNPGL Registry’s secondary aim is to facilitate collaboration among diverse medical disciplines involved in the treatment of HNPGLs. These specialties include but are not limited to vascular surgeons, head and neck surgeons, otolaryngologists, internal medicine specialists such as endocrinologists, clinical geneticists, radiologists, and nuclear physicians.

## Materials and methods

### Status and timeline of the HNPGL Registry

The HNPGL Registry was approved by the medical ethical committee in July 2023 (ID 22–008). The registry is designed and implemented according to the principles of the International Council for Harmonization (ICH) Good Clinical Practice (GCP) guideline and according to the European General Data Protection Regulation (GDPR) [20, 21].

Once local experience has been gained with the inclusion of HNPGL patients in the registry, we intend ‐ by mutual agreement ‐ to collaborate with national and international colleagues to build and implement a large and uniform dataset utilizing standard reporting criteria and a uniform case report form (CRF) for optimal phenotype data analysis.

### Study design and objectives

The HNPGL Registry was designed as a prospective longitudinal and observational registry for extensive data collection on HNPGL patients and carriers of genetic variants causative of HNPGLs. Participation does not influence local protocols and patient management. As this registry is set up in the same research group as the Thoracic Outlet Syndrome (TROTS) registry, the applied protocol format of both registries is almost identical [22]. The HNPGL Registry is initiated by the Department of Vascular Surgery, the Department of Otolaryngology and the Department of Head and Neck Surgical Oncology of the University Medical Center Utrecht (UMCU), a tertiary referral university hospital in the Netherlands. The HNPGL Registry is overseen by a committee from members of both the Department of Vascular Surgery and the Department of Head and Neck Surgical Oncology of the UMCU. The committee is further extended with members of the Department of Endocrine Oncology, Department of Clinical Genetics, Department of Otolaryngology and Department of Radiology and Nuclear Medicine of the UMCU. Patients can be referred to our tertiary center at all times. Patients will be discussed in a multidisciplinary expert panel consisting of a vascular surgeon, head and neck surgeon, endocrinologist, clinical geneticist, radiologist, and nuclear physician.

All patients must give informed consent (IC) before inclusion in the registry, in line with the Dutch law and regulations (‘Wet medisch-wetenschappelijk onderzoek met mensen’) [23]. Upon inclusion, patients are made aware of the PROMs as part of the registry. The primary objective of the registry is to include as many patients as possible, hence the process of inclusion and follow-up will be continuously and indefinitely ongoing.

### Registry population

All patients with a HNPGL, regardless of treatment received, can be included in the registry. Additionally, individuals with a known (likely) pathogenic variant (e.g., but not limited to: *SDHx*, *VHL*, *MEN*, *NF*, *TMEM 127* and *RET*) associated with HNPGLs are eligible for inclusion. Moreover, subjects must be 16 years or older, must be able to read and comprehend the patient information folder (PIF) and IC form, and willing to provide their signed consent (Table 1).

**Table 1:** In- and exclusion criteria for the HNPGL Registry.

**Inclusion criteria:**
• A positive diagnosis for HNPGL(s) and/or a (likely) pathogenic variant in one of the PGL predisposition genes.
• Patient aged 16 years or older.
• Patient and/or their legal representative signed informed consent.
**Exclusion criteria:**
• Subject and/or legal representative who is unable or unwilling to sign informed consent

HNPGL = head and neck paraganglioma; PGL = paraganglioma

### Recruitment and consent process [22]

Individuals eligible for inclusion are identified by the attending physician and the local principal investigator (PI). A PIF and IC form (S1 File) will be provided to the participant as well as verbal information by the local PI at the first hospital visit. The patient obtains a reflection period of at least 24 hours upon dating and signing the IC form by both the individual and the local PI. A unique study ID will be generated by the electronic case report form (eCRF) Castor upon inclusion in the HNPGL Registry. Participants have the right to withdraw from the registry at any time without providing a reason and without facing any consequences. Data will stay included in the registry upon withdrawal, unless specifically mentioned by the participant, to prevent for bias.

### Data collection and monitoring

Castor will be used as eCRF to collect all relevant clinical information regarding the patient’s diagnosis, treatment, and follow-up. Data will be manually extracted from the patient’s electronic medical record. Castor is a browser-based, metadata-driven electronic data capture (EDC) tool for building and managing online databases conform GCP standards [24]. Clinical data will be collected at baseline (time of inclusion) and during follow-up.

In addition, a survey containing the EuroQol 5D-5L (EQ-5D-5L), European Organisation for Research and Treatment of Cancer Quality of Life Questionnaire (EORTC QLQ-C30), Modified Fatigue Impact Scale (MFIS), Short QUestionnaire to Assess Health-enhancing physical activity (SQUASH), Cancer Worry Scale (CWS), and Hospital Anxiety and Depression Scale (HADS) will be sent via Castor by email to the participant [25–30]. This survey will be sent automatically at set points in time, namely: at time of inclusion, one and two years after inclusion, and every five years after inclusion. When patients undergo intervention, the questionnaire will also be sent out.

Table 2 contains an overview of the collected parameters. The complete eCRF can be found in the S2 File. Depending on new insights on HNPGLs these endpoints may be altered and updated. Furthermore, each participating center has the opportunity, upon mutual approval, to add or modify parameters to the dataset. When needed a research protocol amendment will be made and presented to the medical ethical committee. The local PI is responsible for the adequateness, accurateness, and completeness of the data entered in the eCRF. The progress and results of the HNPGL Registry will be made publicly available via presentations during scientific meetings and by publication in medical journals.

**Table 2:** Overview of the main collected parameters of the HNPGL Registry.

**Patient demographics, characteristics, family history, and medical history, including:**
• Age, year of birth, country of origin. • Medical history. • Family history.
**Initial clinical assessment including the anamnesis and physical exam, including:**
• Date of onset of symptoms. • Specifics about referral to a tertiary referral center.
**Diagnostic modalities used and their results:**
• Laboratory tests including 3-methoxytyramine, metanephrine and normetanephrine. • Results from urinary tests including (nor)metanephrine, (nor)adrenaline and dopamine. • Results from laryngoscopy and otoscopy. • Results of DNA panel testing in blood. • (Dynamic) (duplex) ultrasound, CT, MRI, and PET scans.
**Treatment(s) performed, characteristics and outcomes, including:**
• Conservative measurements. • Surgical treatment. • Endovascular interventions. • Radiotherapy. • Systemic therapy. • Peptide Receptor Radionuclide Therapy (PRRT). • Pathology reports including immunohistochemistry (IHC) staining (for SDHA and D).
**Any treatment related complications, including:**
• Short (<30 days) and long term (> 30 days) complications related to surgery. • Short (<30 days) and long term (> 30 days) complications related to endovascular treatment. • Short (<30 days) and long term (> 30 days) complications related to radiotherapy. • Short (<30 days) and long term (> 30 days) complications related to systemic therapy. • Short (<30 days) and long term (> 30 days) complications related to PRRT.
**Follow-up data, if applicable, including:**
• Symptoms during follow-up. • Physical exam during follow-up. • Diagnostics performed during follow-up and detailed description of results. • Change of treatment strategy, including discontinuation of wait-and-scan. • Changes in health-status as a diagnosis of malignancy, or any other disease. • New diagnosis of other paragangliomas.
**Follow-up survey sent at set time points during follow-up, including:**
• EuroQol 5D-5L (EQ-5D-5L). • European Organisation for Research and Treatment of Cancer Quality of Life Questionnaire (EORTC QLQ-C30). • Modified Fatigue Impact Scale (MFIS). • Short QUestionnaire to Assess Health-enhancing physical activity (SQUASH). • Cancer Worry Scale (CWS). • Hospital Anxiety and Depression Scale (HADS).

CT = computed tomography; HNPGL = head and neck paraganglioma; MRI = magnetic resonance imaging; PET = positron emission tomography; PRRT = peptide receptor radionuclide therapy; SDHA = succinate dehydrogenase gene A; SDHD = succinate dehydrogenase gene D.

### Ethical and safety considerations

In accordance with the ICH GCP guideline and General Data Protection Regulation (GDPR) protocols, the HNPGL Registry has been meticulously designed and implemented. The executed IC forms will be securely archived at specified locations within each participating center. These secure repositories house a key file containing comprehensive personal information pertaining to all participants. Rigorous confidentiality protocols are consistently enforced, precluding the disclosure of participant information to external entities. Authorized personnel with access to the key file include the local PI, coordinating researcher, data manager, and, when deemed necessary, government healthcare inspection authorities. To ensure research findings’ reproducibility and facilitate data comprehension and reuse, all modifications to raw data and analytical procedures are to be documented in syntaxes and reflected in database updates by the research team. The research data, encompassing raw data and syntaxes, must be archived by the research team for a duration of 15 years following data publication. The responsibility for disseminating results lies with the research team, who must submit their findings to a peer-reviewed journal. Upon paper publication, the HNPGL Registry data used will be made publicly available without restrictions in a designated repository. Published papers must reference this registry protocol.

In case of expansion outside the European Union (EU), specific permission for encrypted, pseudonymized data sharing with participating centers outside the EU must be signed, according to the European GDPR. Participants may choose to decline this which will be documented in the eCRF (S2 File). Such data requests by participating centers outside the EU will only be honored if comparable legislation is in place in the respective country.

## Discussion and conclusion

The field of HNPGL research is hindered by a lack of high-quality data, leading to varied recommendations in guidelines, which may even contradict each other. This is partially due to the challenges posed by the rarity of HNPGLs and the involvement of multiple medical specialties. Prior investigations have predominantly relied on retrospective, single-center, and single-discipline datasets. In response to these limitations, the HNPGL Registry seeks to establish a prospective, multicenter, and multidisciplinary research consortium to systematically collect standardized data from individuals afflicted with HNPGL(s) and/or carriers of genetic variants causative of HNPGLs. By utilizing the HNPGL Registry data, researchers can examine various research questions and improve the understanding of optimal management strategies for HNPGLs. This initiative lays the groundwork for subsequent research endeavors and the development of comprehensive guidelines.

## Supporting information

S1 File The patient information form and informed consent form of the Head and Neck Paraganglioma (HNPGL) Registry. (PDF)

S2 File The HNPGL Registry electronic case report form. (PDF)

S3 File The HNPGL Registry protocol version 8.0 as approved by the medical ethical review board Utrecht, The Netherlands. (PDF)
