# Supplementary material for: Head and Neck Paraganglioma (HNPGL) Registry: A study protocol for prospective data collection in patients with Head and Neck Paragangliomas
Source: PLoS One. 2024 Jul 25;19(7):e0307311. doi: 10.1371/journal.pone.0307311 (PMC11271953; doi:10.1371/journal.pone.0307311)
Supplement: S1 File — (PDF) [file pone.0307311.s001.pdf]

Proefpersoneninformatie voor deelname aan een registratie voor medisch-wetenschappelijk onderzoek

Titel: Hoofd-/halsparagangliomen database

Engels: Head and Neck Paragangliomas Registry

Inleiding

Geachte mevrouw, meneer,

In deze brief vragen we u of u wilt meewerken aan de bovengenoemde registratie (zie titel) waarin gegevens worden verzameld voor toekomstig medisch-wetenschappelijk onderzoek. Meedoen is vrijwillig. Om mee te doen is wel uw toestemming nodig. U ontvangt deze brief omdat u een paraganglioom in het hoofdhalsgebied heeft of omdat u een genetische afwijking hebt welke kan leiden tot een paraganglioom in het hoofdhalsgebied

U leest hier wat het voor u betekent, en wat de voordelen en nadelen zijn. Het is veel informatie. Wilt u de informatie doorlezen en beslissen of u wilt meedoen? Als u wilt meedoen, kunt u het formulier invullen dat u vindt in bijlage 2.

Stel uw vragen

U kunt uw beslissing nemen met de informatie die u in deze informatiebrief vindt. Daarnaast raden we u aan om dit te doen:

- Stel vragen aan de onderzoeker die u deze informatie geeft.
- Praat met uw partner, familie of vrienden over dit onderzoek.

1. Algemene informatie

Dit onderzoek is een samenwerking van meerdere ziekenhuizen in Nederland, waaronder het UMC Utrecht. Het onderzoek wordt gecoördineerd en beheerd door het Universitair Medisch Centrum Utrecht (UMC Utrecht).

Voor een academisch ziekenhuis is het van groot belang dat (medische) gegevens van patiënten voor medisch wetenschappelijk onderzoek wordt verzameld. De (medische) gegevens worden opgeslagen. Dit noemen we een registratie. Op het moment van verzamelen is nog niet bekend voor welk specifiek onderzoek de gegevens zullen worden gebruikt. In het

algemeen gaat het om onderzoek naar oorzaken van ziekten, onderzoek dat kan leiden tot betere diagnose van ziekten, onderzoek waardoor ziekten beter kunnen worden voorspeld en onderzoek dat kan bijdragen aan de ontwikkeling van betere en/of nieuwe behandelingen.
Het UMC Utrecht heeft dit onderzoek opgezet. Hieronder noemen we het UMC Utrecht steeds de ‘opdrachtgever’. Onderzoekers, dit kunnen zowel artsen, als onderzoeker zijn, voeren het onderzoek uit in dit ziekenhuis.
In het algemeen gaat het om onderzoek naar oorzaken van ziekte:

- onderzoek dat kan leiden tot betere diagnose van ziekten
- onderzoek waardoor ziekten beter kunnen worden voorspeld
- onderzoek dat kan bijdragen aan de ontwikkeling van nieuwe behandelingen.

2. Doel van het onderzoek

Voor toekomstig onderzoek naar hoofdhals paragangliomen willen wij medische gegevens en van patiënten met een hoofd-/halsparaganglioom en/of een genetische afwijking die kan leiden tot een paraganglioom in het hoofdhals gebied. Gezien het zeldzame karakter van hoofdhals paragangliomen is het streven van deze registratie om nationaal en uiteindelijk ook internationaal patiënten te vragen mee te doen om zo zoveel mogelijk gegevens te verzamelen.
Gegevens die wij verzamelen in deze registratie zijn:

- Uw genetische mutatie
- Uitslagen van diagnostische onderzoeken
- Uitkomst van eventuele behandeling(en)
- Informatie middels een vragenlijst over de kwaliteit van leven

3. Wat meedoen inhoudt

Als u deelneemt aan deze registratie dan vragen wij van u het volgende:

1. Toestemming aan het UMC Utrecht om (medische) gegevens te verzamelen, zoals :
   - Enkele basisgegevens van de patiënt: geslacht, leeftijd, voorgeschiedenis, etc.
   - Genetisch onderzoek
   - Basisgegevens t.a.v. het ziektebeeld: aangedane zijde, klachtenpatroon, eventuele beeldvorming, etc.
   - Gegevens t.a.v. de behandeling: medicatie, ingrepen en operaties.
   - Uitkomst van de behandeling, waaronder vragenlijsten.

   In het kader van deze registratie worden uw gegevens in de toekomst bewaard en gebruikt voor de doelstellingen beschreven in de informatiebrief.

2. Toestemming voor het versturen van vragenlijsten, zoals vermeld in de informatiebrief, om de kwaliteit van leven met mensen met deze aandoening te meten. Deze vragenlijsten zullen elektronisch worden verstuurd naar uw e-mailadres, direct nadat u besluit mee te doen, daarna na 1, 2 en 5 jaar en vervolgens elke 5 jaar. De vragenlijsten worden verstuurd met het programma Castor, Castor is een online systeem waarin het UMC Utrecht de gegevens verzamelt. Het invullen van deze vragenlijsten zal ongeveer 15 minuten per keer in beslag nemen. Deze vragenlijsten zijn niet onderdeel van de ‘standaard zorg’; deze zijn dus puur in het kader van deze registratie.

3. Toestemming om u te benaderen voor het verstrekken van extra gegevens, indien dit voor een bepaald wetenschappelijk onderzoek noodzakelijk is.
Als u hier 'nee' antwoordt, zal dit bij uw gegevens worden genoteerd.

4. Toestemming om gegevens op te vragen bij de Basisregistratie Personen.
Om in de toekomst op de hoogte te blijven van de juiste persoonsgegevens, vragen wij uw toestemming om, zo nodig, van de gemeente waar u woont informatie uit de Basisregistratie op te vragen. Vanzelfsprekend respecteren wij het als u hiervoor geen toestemming geeft. De persoonsgegevens hebben wij bijvoorbeeld nodig om u te kunnen informeren in geval van bevindingen zoals wij later in deze brief uitleggen. Als u geen toestemming geeft om in de toekomst gegevens op te vragen bij de Basisregistratie Personen kan dat betekenen dat wij mogelijk in het geval van een bevinding geen contact met u op kunnen nemen.

5. Toestemming om uw medische gegevens op te vragen en te gebruiken.
Met medische gegevens uit uw behandeldossier nu en in de toekomst kunnen wij de oorzaken en complicaties van verschillende ziekten beter bestuderen. Dit geldt ook eventueel voor medische gegevens uit behandeldossiers van uw huisarts of van andere ziekenhuizen als u daar onder behandeling bent (geweest). Uw medische gegevens worden altijd in gecodeerde vorm door de datamanager van de registratie beschikbaar gesteld (dus onherkenbaar gemaakt) voordat ze gebruikt worden door de onderzoeker. Op die manier zorgen we ervoor dat de onderzoeker uw identiteit niet te weten kan komen.

6. Toestemming om gegevens op te vragen m.b.t. de doodsoorzaak bij het Centraal Bureau voor de Statistiek (CBS). Het CBS is de officiële instantie in Nederland die doodsoorzaken registreert.
Mocht u komen te overlijden, dan zouden wij graag bij het CBS navraag willen doen over uw doodsoorzaak.

De bij u verzamelde (medische) gegevens blijven onbeperkt ter beschikking voor onderzoek, tenzij u uw toestemming intrekt. Mocht u onverhoopt komen te overlijden dan blijft uw toestemming onverkort van kracht en uw nabestaanden hebben daar geen zeggenschap over.

7. Toestemming om uw medische gegevens die wij verzameld hebben, geanonimiseerd, te delen met andere deelnemende ziekenhuizen, inclusief ziekenhuizen buiten Nederland, voor verder onderzoek naar uw aandoening.

8. De bij u verzamelde (medische) gegevens blijven onbeperkt ter beschikking voor onderzoek, tenzij u uw toestemming intrekt. Mocht u onverhoopt komen te overlijden dan blijft uw toestemming onverkort van kracht en uw nabestaanden hebben daar geen zeggenschap over.

4. Mogelijke voor- en nadelen

Deelname aan deze registratie levert voor uzelf geen direct voordeel op. De onderzoeksresultaten worden in principe niet aan u teruggekoppeld. Wel kunnen uitkomsten van het onderzoek uw zorg en die van andere mensen met vergelijkbare aandoeningen in de toekomst verbeteren.
Een mogelijk nadeel van de vragenlijsten is dat het tijd kost om deze in te vullen en terug te sturen.

5. Als u niet wilt meedoen of wilt stoppen met deze registratie

U beslist zelf of u meedoet aan deze registratie. Deelname is vrijwillig. Als u niet wilt meedoen, krijgt u gewoon dezelfde behandeling als anders.
Als u wel meedoet, kunt u altijd zelf stoppen met het onderzoek. Dat mag op ieder moment. Meld dit dan meteen bij de onderzoeker. U hoeft er niet bij te vertellen waarom u stopt.
U kunt dit melden door het intrekkingsformulier in de bijlage op te sturen aan het hoofd van de afdeling Vaatchirurgie van het UMC Utrecht.

6. Gebruik van uw gegevens

Voor deze registratie worden uw persoonsgegevens verzameld, gebruikt en bewaard. Het gaat om gegevens zoals uw naam, geboortedatum en om gegevens over uw gezondheid. Het verzamelen, gebruiken en bewaren van uw gegevens is nodig om de vragen voor toekomstig medisch-wetenschappelijk onderzoek te kunnen beantwoorden en de resultaten te kunnen publiceren. Het UMC Utrecht verzamelt uw gegevens en deelt deze vervolgens op een gepseudonimiseerde manier, dat wil zeggen niet direct tot u herleidbaar, met de beheerder van het onderzoek; het UMC Utrecht. Onderzoek met uw gegevens zal in principe alleen worden uitgevoerd onder verantwoordelijkheid van in het UMC Utrecht aangestelde onderzoekers. Het onderzoek kan eventueel ook worden uitgevoerd in samenwerking met andere (buitenlandse) ziekenhuizen waarbij dan altijd een in het UMC Utrecht aangestelde onderzoeker betrokken blijft. Voor dergelijk onderzoek kan het nodig zijn om de (medische) gegevens te verstrekken aan deze ziekenhuizen. Dit zal echter altijd gebeuren op een manier dat de gegevens niet tot u zijn te herleiden.

In rapporten en publicaties over het onderzoek zijn de gegevens eveneens niet tot u te herleiden. Bij het verwerken van uw gegevens houden wij ons aan de Algemene Verordening Gegevensbescherming. Wat dit precies voor u betekend kunt u lezen in de bijlage. Wij vragen voor het gebruik van uw gegevens uw toestemming.

Waarom verzamelen, gebruiken en bewaren we uw gegevens?
We verzamelen, gebruiken en bewaren uw gegevens om in de toekomst vragen over paragangliomen te kunnen beantwoorden en deze resultaten te kunnen publiceren.
Bij het verwerken van uw gegevens houden wij ons aan de Algemene Verordening Gegevensbescherming. Wat dit precies voor u betekent kunt u lezen in de bijlage. Wij vragen voor het gebruik van uw gegevens uw toestemming.

7. Geen vergoeding voor meedoen

Voor het meedoen aan deze registratie krijgt u geen onkostenvergoeding.

8. Heeft u vragen?

Vragen over deze registratie kunt u stellen aan uw behandelend arts of de coördinerend arts-onderzoeker: B.J. Petri b.j.petri@umcutrecht.nl of J.M. de Bresser j.m.debresser-4@umcutrecht.nl

Heeft u een klacht? Bespreek dit dan met de onderzoeker of de arts die u behandelt. Wilt u dit liever niet? Ga dan naar de klachtenbemiddelaars. Deze zijn bereikbaar via tel. 088-75 562 08. Of digitaal via: http://www.umcutrecht.nl/nl/Ziekenhuis/Ervaringen-van-patienten/Een-klacht-indienen.

9. Ondertekening toestemmingsformulier

U kunt eerst rustig nadenken over deze registratie. Daarna vertelt u de onderzoeker of u de informatie begrijpt en of u wel of niet wilt meedoen. Wilt u meedoen? Dan vult u het toestemmingsformulier in dat u bij deze informatiebrief vindt. U en de onderzoeker krijgen allebei een getekende versie van deze toestemmingsverklaring.

Dank voor uw tijd.

Met vriendelijke groet, namens de Head and Neck Paraganglioma Registry

Prof. dr. G.J. de Borst (hoogleraar vaatchirurgie UMC Utrecht)
tel.: 088-7556965

Bijlagen:

1. Aanvullende informatie over verwerking van uw gegevens
2. Toestemmingsformulier
3. Intrekkingsformulier

Bijlage 1: Aanvullende informatie over verwerking van uw gegevens

Hoe beschermen we uw privacy?
Om uw privacy te beschermen geven wij uw gegevens een code. De sleutel van de code bewaren we op een beveiligde plek in de lokale onderzoeksinstelling. Als we uw gegevens verwerken, gebruiken we steeds alleen die code. Ook in rapporten en publicaties over het onderzoek kan niemand terughalen dat het over u ging.

Wie kunnen uw gegevens zien?
Sommige personen kunnen wel uw naam en andere persoonlijke gegevens zonder code inzien. Dit zijn mensen die controleren of de onderzoekers het onderzoek goed en betrouwbaar uitvoeren. Deze personen kunnen bij uw gegevens komen:

- Een controleur die voor de onderzoeker werkt.
- Nationale toezichthoudende autoriteiten.

Deze personen houden uw gegevens geheim. Wij vragen u voor deze inzage toestemming te geven. De Inspectie Gezondheidszorg en Jeugd kan zonder uw toestemming uw gegevens inzien.

Hoelang bewaren we uw gegevens?
Uw gegevens worden voor onbepaalde tijd bewaard op de onderzoekslocatie.

Kunt u uw toestemming ook weer intrekken?
U kunt uw toestemming voor gebruik van uw persoonsgegevens altijd weer intrekken.
Indien u uw toestemming intrekt, houdt dat in dat er geen nieuwe (medische) gegevens verzameld worden. Daarnaast kunt u kiezen uit twee mogelijkheden:

1) De tot dan toe verzamelde (medische) gegevens blijven beschikbaar voor wetenschappelijk onderzoek zoals vastgelegd in het toestemmingsformulier.

2) U verzoekt dat er geen (medische) gegevens meer gebruikt worden ten behoeve van onderzoek met deze registratie.
Indien u dat expliciet aangeeft zullen alle (medische) gegevens dat van u is verzameld voor deze registratie worden vernietigd, behalve de gegevens die gebruikt zijn in een wetenschappelijk onderzoek.

We sturen uw gegevens naar landen buiten de Europese Unie
In dit onderzoek sturen we mogelijk uw gecodeerde gegevens ook naar landen buiten de Europese Unie. In die landen gelden niet de privacyregels van de Europese Unie. Wij vragen hiervoor uw toestemming.

Wilt u meer weten over uw privacy?

- Wilt u meer weten over uw rechten bij de verwerking van persoonsgegevens? Kijk dan op www.autoriteitpersoonsgegevens.nl.

- Heeft u vragen over uw rechten? Of heeft u een klacht over de verwerking van uw persoonsgegevens? Neem dan contact op met degene die verantwoordelijk is voor de verwerking van uw persoonsgegevens. Voor uw onderzoek is dat:
    - Dr. B.J. Petri, vaatchirurg UMC Utrecht
      www.umcutrecht.nl/nl/verwijsprocedure/vaatchirurgie
- Als u klachten heeft over de verwerking van uw persoonsgegevens, raden we u aan om deze eerst te bespreken met het onderzoeksteam. U kunt ook contact opnemen met de Functionaris Gegevensbescherming van het UMC Utrecht gaan via privacy@umcutrecht.nl. Of u dient een klacht in bij de Autoriteit Persoonsgegevens.

Bijlage 2: Toestemmingsformulier proefpersoon

Head and Neck Paraganglioma Registry

- Ik heb de informatiebrief gelezen. Ook kon ik vragen stellen. Mijn vragen zijn voldoende beantwoord. Ik had genoeg tijd om te beslissen of ik meedoe.

- Ik weet dat meedoen vrijwillig is. Ook weet ik dat ik op ieder moment kan beslissen om toch niet mee te doen of te stoppen met de registratie. Daarvoor hoef ik geen reden te geven.

- Ik geef toestemming voor het verzamelen en gebruiken van mijn (medische) gegevens voor wetenschappelijk onderzoek op het gebied van hoofdhals paragangliomen zoals beschreven in de informatiebrief.

- Ik geef toestemming om de verzamelde (medische) gegevens voor onbepaalde tijd te bewaren voor toekomstig wetenschappelijk onderzoek voor de doelstellingen beschreven in de informatiebrief.

- Ik weet dat voor de controle van deze registratie sommige mensen toegang tot al mijn gegevens kunnen krijgen. Die mensen staan vermeld in de bijlage bij deze informatiebrief. Ik geef toestemming voor die inzage door deze personen.

- Ik geef toestemming voor het doorsturen in het kader van deze registratie van mijn gegevens. Gegevens moeten gecodeerd worden overgedragen en zonder mijn naam en andere persoonlijke gegevens die mij direct kunnen identificeren.

- Ik weet dat mijn gecodeerde gegevens naar landen buiten de EU worden gestuurd waar privacyregels van de EU niet gelden. Ik geef hiervoor toestemming.

- Ik geef toestemming dat de vragenlijst wordt verstuurd naar mijn emailadres. De vragenlijsten worden verstuurd vanuit en naar het programma Castor.

- Ik geef ☐ **wel**
  ☐ **geen** toestemming om mij na dit onderzoek opnieuw te benaderen voor een vervolgonderzoek.

- Ik geef ☐ **wel**
  ☐ **geen** toestemming om te worden benaderd voor het verstrekken van extra gegevens, indien dit voor een bepaald onderzoek noodzakelijk is

- Ik geef ☐ **wel**
  ☐ **geen** toestemming om zo nodig in de toekomst mijn persoonsgegevens op te vragen bij de Basisregistratie Personen.

- Ik geef □ **wel**

  □ **geen** toestemming om zo nodig in de toekomst medische informatie op te vragen bij mijn huisarts en andere ziekenhuizen waar ik ben behandeld.

- Ik geef □ **wel**

  □ **geen** toestemming om zo nodig in de toekomst mijn doodsoorzaak op te vragen bij het CBS

- Ik geef □ **wel**

  □ **geen** toestemming om de gecodeerde gegevens door te sturen naar landen buiten de EU waar de EU-regels ter bescherming van de persoonsgegevens van deze persoon niet gelden

- Ik wil meedoen aan dit onderzoek.

Naam proefpersoon:

Handtekening: Datum : __ / __ / __

Ik verklaar dat ik deze proefpersoon volledig heb geïnformeerd over het genoemde onderzoek.

Als er tijdens het onderzoek informatie bekend wordt die de toestemming van de proefpersoon zou kunnen beïnvloeden, dan breng ik hem/haar daarvan tijdig op de hoogte.

Naam onderzoeker (of diens vertegenwoordiger):

Handtekening: Datum: __ / __ / __

----

* Doorhalen wat niet van toepassing is.

De proefpersoon krijgt een volledige informatiebrief mee, samen met een kopie van het getekende toestemmingsformulier.

Bijlage 2: Toestemmingsformulier vertegenwoordiger

Head and Neck Paraganglioma Registry

Ik ben gevraagd om toestemming te geven voor deelname van de volgende persoon aan deze registratie:

Naam proefpersoon: Geboortedatum: __ / __ / __

- Ik heb de informatiebrief gelezen. Ook kon ik vragen stellen. Mijn vragen zijn voldoende beantwoord. Ik had genoeg tijd om te beslissen of ik wil dat deze persoon meedoet.

- Ik weet dat meedoen vrijwillig is. Ook weet ik dat ik op ieder moment kan beslissen dat deze persoon toch niet meedoet of om te stoppen met de registratie. Daarvoor hoef ik geen reden te geven.

- Ik geef toestemming voor het verzamelen en gebruiken van de (medische) gegevens van deze persoon voor wetenschappelijk onderzoek op het gebied van hoofdhals paragangliomen zoals beschreven in de informatiebrief.

- Ik geef toestemming om de verzamelde (medische) gegevens van deze persoon voor onbepaalde tijd te bewaren voor toekomstig wetenschappelijk onderzoek voor de doelstellingen beschreven in de informatiebrief.

- Ik weet dat voor de controle van deze registratie sommige mensen toegang tot de gegevens van deze persoon kunnen krijgen. Die mensen staan vermeld in de bijlage bij deze informatiebrief. Ik geef toestemming voor die inzage door deze personen.

- Ik geef toestemming voor het doorsturen in het kader van deze registratie van de gegevens van deze persoon. Gegevens moeten gecodeerd worden overgedragen en zonder naam en andere persoonlijke gegevens die deze persoon direct kunnen identificeren.

- Ik weet dat mijn gecodeerde gegevens naar landen buiten de EU worden gestuurd waar privacyregels van de EU niet gelden.

- Ik geef toestemming dat de vragenlijst wordt verstuurd naar mijn emailadres. De vragenlijsten worden verstuurd vanuit en naar het programma Castor.

- Ik geef □ **wel**
□ **geen** toestemming om deze persoon opnieuw te benaderen voor een vervolgonderzoek.

- Ik geef □ **wel**

  □ **geen** toestemming om te worden benaderd voor het verstrekken van extra gegevens, indien dit voor een bepaald onderzoek noodzakelijk is

- Ik geef □ **wel**

  □ **geen** toestemming om zo nodig in de toekomst de persoonsgegevens van deze persoon op te vragen bij de Basisregistratie Personen.

- Ik geef □ **wel**

  □ **geen** toestemming om zo nodig in de toekomst medische informatie op te vragen bij de huisarts en andere ziekenhuizen waar deze persoon is behandeld.

- Ik geef □ **wel**

  □ **geen** toestemming om zo nodig in de toekomst de doodsoorzaak van deze persoon op te vragen bij het CBS

- Ik geef □ **wel**

  □ **geen** toestemming om de gecodeerde gegevens door te sturen naar landen buiten de EU waar de EU-regels ter bescherming van de persoonsgegevens van deze persoon niet gelden

- Ik ga ermee akkoord dat deze persoon meedoet aan deze registratie.

Naam wettelijk vertegenwoordiger:
Relatie tot de proefpersoon:
Handtekening: Datum: __ / __ / __

Ik verklaar hierbij dat ik deze persoon/personen volledig heb geïnformeerd over het genoemde onderzoek.

Als er tijdens het onderzoek informatie bekend wordt die de toestemming van de wettelijk vertegenwoordiger zou kunnen beïnvloeden, dan breng ik hem/haar daarvan tijdig op de hoogte.

Naam onderzoeker (of diens vertegenwoordiger):
Handtekening: Datum: __ / __ / __

----

* Doorhalen wat niet van toepassing is.

De vertegenwoordiger krijgt een volledige informatiebrief mee, samen met een kopie van het getekende toestemmingsformulier.

Bijlage 3: Intrekkingsformulier

Head and Neck Paraganglioma Registry

Ik geef hiermee te kennen dat ik mijn deelname aan deze registratie intrek. Dit betekent dat van mij geen nieuwe (medische) gegevens meer mogen worden verzameld en er geen vragenlijsten meer verstuurd worden voor toekomstig onderzoek naar hoofdhals paragangliomen.

Ik begrijp dat de (medische) gegevens die in een onderzoek zijn gebruikt niet worden teruggehaald of vernietigd. Deze (medische) gegevens blijven gecodeerd ter beschikking van degene die het onderzoek uitvoert.

Over het van mij nog opgeslagen lichaamsmateriaal ten behoeve van deze registratie verklaar ik dat mijn lichaamsmateriaal:

O nog steeds gebruikt mag worden volgens het door mij eerder ondertekende toestemmingsformulier,
O vernietigd moet worden.

Naam: ______________________________

Geboortedatum: ______________

Datum: Handtekening:

Formulier opsturen naar hoofd van de afdeling Vaatchirurgie:

Ik verklaar kennis genomen te hebben van het intrekken van de toestemming door de boven vermelde patiënt en zoals hierboven omschreven.

Instelling: UMC Utrecht

Naam afdelingshoofd: ______________________________

Datum: ____________ Handtekening ______________________
