# Supplementary material for: Head and Neck Paraganglioma (HNPGL) Registry: A study protocol for prospective data collection in patients with Head and Neck Paragangliomas
Source: PLoS One. 2024 Jul 25;19(7):e0307311. doi: 10.1371/journal.pone.0307311 (PMC11271953; doi:10.1371/journal.pone.0307311)
Supplement: S2 File — (PDF) [file pone.0307311.s002.pdf]

Head and Neck Paraganglioma Registry - version 233.91

Printed on 06-12-2023 19:29:31 by Carolijn de Bresser

1. Inclusion - Informed Consent

Number	Question	Answers
1.1	Signed Informed Consent?<br>*Exclude patient if field's value is equal to No with message: 'Informed Consent must be signed.'*	◯ Yes<br>◯ No
1.2	Date of signed informed consent<br>*Exclude patient if field's value is larger than NOW with message: 'Date cannot be in the future.'*	☐ ☐ ☐ *(dd-mm-yyyy)*
1.3	Date of diagnosis<br>*Warning shown if field's value is larger than NOW: 'Date of diagnosis can't be in the future'*	☐ ☐ ☐ *(dd-mm-yyyy)*

2. Inclusion - Inclusion Criteria

Number	Question	Answers
2.1	Is patient ≥ 16 years upon diagnosis?	◯ Yes<br>◯ No
2.2	Does the patient have/had HNPGL(s)?	◯ Yes<br>◯ No
2.3	Does the patient have a known genetic mutation	◯ Yes<br>◯ No<br>◯ Unknown
2.3.1	***If 'Does the patient have a known genetic mutation' is equal to 'Yes' answer this question:***<br>What type of mutation	☐ SDHA<br>☐ SDHB<br>☐ SDHC<br>☐ SDHD<br>☐ SDHAF2<br>☐ VHL<br>☐ NF1<br>☐ TMEM127<br>☐ MAX<br>☐ Unknown<br>☐ Other
2.3.1.1	***If 'What type of mutation' is equal to 'Other' answer this question:***<br>What other mutation	
2.3.2	***If 'Does the patient have a known genetic mutation' is equal to 'Unknown' answer this question:***<br>Why is the mutation unknown?	◯ Genetic research follows<br>◯ Patient doesn't want genetic research<br>◯ Other
2.3.2.1	***If 'Why is the mutation unknown?' is equal to 'Other' answer this question:***<br>What other reason?	
2.4	Does the patient meet the inclusion criteria?	

3. Patient characteristics - Demographics

Number	Question	Answers
3.1	Year of birth	☐ *(yyyy)*
3.2	Gender	○ Female<br>○ Male
3.2.1	***If 'Gender' is equal to 'Female' answer this question:***<br>Does the patient have children via birth?	☐ Yes<br>☐ No<br>☐ Unknown
3.2.2	***If 'Gender' is equal to 'Female' answer this question:***<br>Is the patient pregnant at this moment?	☐ Yes<br>☐ No<br>☐ Unknown
3.2.3	***If 'Gender' is equal to 'Female' answer this question:***<br>Does the patient have/had a pregnancy wish?	☐ Yes<br>☐ No<br>☐ Unknown

3.3 Country of origin

- ◯ Afghanistan
- ◯ Albania
- ◯ Algeria
- ◯ Andorra
- ◯ Angola
- ◯ Antigua and Barbuda
- ◯ Argentina
- ◯ Armenia
- ◯ Australia
- ◯ Austria
- ◯ Azerbaijan
- ◯ Bahamas
- ◯ Bahrain
- ◯ Bangladesh
- ◯ Barbados
- ◯ Belarus
- ◯ Belgium
- ◯ Belize
- ◯ Benin
- ◯ Bhutan
- ◯ Bolivia
- ◯ Bosnia and Herzegovina
- ◯ Botswana
- ◯ Brazil
- ◯ Brunei
- ◯ Bulgaria
- ◯ Burkina Faso
- ◯ Burundi
- ◯ Cabo Verde
- ◯ Cambodia
- ◯ Cameroon
- ◯ Canada
- ◯ Central African Republic
- ◯ Chad
- ◯ Chile
- ◯ China
- ◯ Colombia
- ◯ Comoros
- ◯ Democratic Republic of the Congo
- ◯ Republic of the Congo
- ◯ Costa Rica
- ◯ Cote d Ivoire
- ◯ Croatia
- ◯ Cuba
- ◯ Cyprus
- ◯ Czech Republic

- ◯ Denmark
- ◯ Djibouti
- ◯ Dominica
- ◯ Dominican Republic
- ◯ Ecuador
- ◯ Egypt
- ◯ El Salvador
- ◯ Equatorial Guinea
- ◯ Eritrea
- ◯ Estonia
- ◯ Ethiopia
- ◯ Fiji
- ◯ Finland
- ◯ France
- ◯ Gabon
- ◯ Gambia
- ◯ Georgia
- ◯ Germany
- ◯ Ghana
- ◯ Greece
- ◯ Grenada
- ◯ Guatemala
- ◯ Guinea
- ◯ Guinea-Bissau
- ◯ Guyana
- ◯ Haiti
- ◯ Honduras
- ◯ Hungary
- ◯ Iceland
- ◯ India
- ◯ Indonesia
- ◯ Iran
- ◯ Iraq
- ◯ Ireland
- ◯ Israel
- ◯ Italy
- ◯ Jamaica
- ◯ Japan
- ◯ Jordan
- ◯ Kazakhstan
- ◯ Kenya
- ◯ Kiribati
- ◯ Kosovo
- ◯ Kuwait
- ◯ Kyrgyzstan
- ◯ Laos
- ◯ Latvia

- ○ Lebanon
- ○ Lesotho
- ○ Liberia
- ○ Libya
- ○ Liechtenstein
- ○ Lithuania
- ○ Luxembourg
- ○ Macedonia
- ○ Madagascar
- ○ Malawi
- ○ Malaysia
- ○ Maldives
- ○ Mali
- ○ Malta
- ○ Marshall Islands
- ○ Mauritania
- ○ Mauritius
- ○ Mexico
- ○ Micronesia
- ○ Moldova
- ○ Monaco
- ○ Mongolia
- ○ Montenegro
- ○ Morocco
- ○ Mozambique
- ○ Myanmar (Burma)
- ○ Namibia
- ○ Nauru
- ○ Nepal
- ○ Netherlands
- ○ New Zealand
- ○ Nicaragua
- ○ Niger
- ○ Nigeria
- ○ North Korea
- ○ Norway
- ○ Oman
- ○ Pakistan
- ○ Palau
- ○ Palestine
- ○ Panama
- ○ Papua New Guinea
- ○ Paraguay
- ○ Peru
- ○ Philippines
- ○ Poland
- ○ Portugal

- Qatar
- Romania
- Russia
- Rwanda
- Saint Kitts and Nevis
- Saint Lucia
- Saint Vincent and the Grenadines
- Samoa
- San Marino
- Sao Tome and Principe
- Saudi Arabia
- Senegal
- Serbia
- Seychelles
- Sierra Leone
- Singapore
- Slovakia
- Slovenia
- Solomon Islands
- Somalia
- South Africa
- South Korea
- South Sudan
- Spain
- Sri Lanka
- Sudan
- Suriname
- Swaziland
- Sweden
- Switzerland
- Syria
- Taiwan
- Tajikistan
- Tanzania
- Thailand
- Timor-Leste
- Togo
- Tonga
- Trinidad and Tobago
- Tunisia
- Turkey
- Turkmenistan
- Tuvalu
- Uganda
- Ukraine
- United Arab Emirates
- United Kingdom

		◯ United States of America<br>◯ Uruguay<br>◯ Uzbekistan<br>◯ Vanuatu<br>◯ Vatican City<br>◯ Venezuela<br>◯ Vietnam<br>◯ Yemen<br>◯ Zambia<br>◯ Zimbabwe
3.3.1	***If 'Country of origin' is not equal to 'Netherlands' answer this question:***<br>Has patient lived at high altitudes?	☐ Yes<br>☐ No<br>☐ Unknown
3.4	Hypertension in medical history?	◯ Yes<br>◯ No<br>◯ Unknown
3.5	Hypertensive crisis in medical history?	◯ Yes<br>◯ No
3.5.1	***If 'Hypertensive crisis in medical history?' is equal to 'Yes' answer this question:***<br>Organ damage due to hypertensive crisis?	◯ Yes<br>◯ No<br>◯ Unknown
3.6	All other relevant medical history	
3.7	Does patient use any relevant medication?	◯ Yes<br>◯ No<br>◯ Unknown

3.7.1	***If 'Does patient use any relevant medication?' is equal to 'Yes' answer this question:***<br>Medication used	☐ Alpha blockage<br>☐ Beta blockage<br>☐ ACE-inhibitor<br>☐ Benzodiazepines<br>☐ Vasodilators<br>☐ Diuretics<br>☐ Calcium channel antagonoists<br>☐ SSRI (Selective Serotonine Reuptake Inhibitor)<br>☐ TCA (tricyclic antidepressant)<br>☐ MAO inhibitor<br>☐ Anticoagulant<br>☐ Other
3.7.1.1	***If 'Medication used' is equal to 'Other' answer this question:***<br>Other medication used	
3.8	Diagnosed mutation	
3.9	Parent of mutational origin know?	○ Yes<br>○ No
3.9.1	***If 'Parent of mutational origin know?' is equal to 'Yes' answer this question:***<br>Parent of origin	○ Father<br>○ Mother
3.9.2	***If 'Parent of mutational origin know?' is equal to 'Yes' answer this question:***<br>Anamnestic or clinically proven positive family history?	○ Anamnestic<br>○ Clinically proven<br>○ Unknown

3.10	Other clinically affected relative(s) known?	☐ Mother<br>☐ Father<br>☐ Sister<br>☐ Brother<br>☐ Grandfather<br>☐ Grandmother<br>☐ Cousin<br>☐ Son<br>☐ Daughter<br>☐ Second-cousin<br>☐ Great uncle<br>☐ Great aunt<br>☐ Uncle<br>☐ Aunt<br>☐ Other<br>☐ Unknown<br>☐ None
3.10.1	***If 'Other clinically affected relative(s) known?' is equal to 'Other' answer this question:***<br>Other affected family member	
	NOTE: malignant paraganglioma are defined as PGL with distant metastases	
3.11	Malignant paraganglioma in the family?	○ Yes<br>○ No<br>○ Unknown

4. Patient characteristics - First visit

Number	Question	Answers
4.1	First symptoms	☐ Swelling in the neck<br>☐ Hoarseness<br>☐ Pain<br>☐ Difficulty swallowing<br>☐ Tinnitus<br>☐ Hearing loss<br>☐ Palpitations<br>☐ Excessive sweating<br>☐ Incidentaloma<br>☐ Dizziness<br>☐ Visual complaints<br>☐ Coughing<br>☐ Malignant hypertension<br>☐ Agitated<br>☐ No complaints<br>☐ Other<br>☐ Unknown
4.1.1	***If 'First symptoms' is equal to 'No complaints' answer this question:***<br>Screening due to familial mutation follow up?	◯ Yes<br>◯ No
4.1.2	***If 'First symptoms' is equal to 'Other' answer this question:***<br>Other experienced symptoms	
4.2	Year of experiencing first symptoms / diagnosis	____ *(yyyy)*
4.3	Date first PGL hospital visit<br>*Exclude patient if field's value is larger than NOW with message: 'Date cannot be in the future.'*	____ ____ ____ *(dd-mm-yyyy)*

4.4	Type of specialist of first visit	☐ Vascular surgeon<br>☐ Head and neck surgeon<br>☐ Otorhinolaryngologist<br>☐ Endocrinologist<br>☐ Internist<br>☐ Clinical genetics<br>☐ Radiologist<br>☐ Gynaecologist<br>☐ Cardiologist<br>☐ Psychiatrist<br>☐ Neurologist<br>☐ Other
4.4.1	***If 'Type of specialist of first visit' is equal to 'Other' answer this question:***<br>Other specialist	
	NOTE: malignant paraganglioma are defined as PGL with distant metastasize	
4.5	Malignant paraganglioma upon first visit?	○ Yes<br>○ No<br>○ Unknown
4.5.1	***If 'Malignant paraganglioma upon first visit?' is equal to 'Yes' answer this question:***<br>Location of metastasis/metastases	☐ Locoregional<br>☐ Distant
4.5.1.1	***If 'Location of metastasis/metastases' is equal to 'Locoregional' answer this question:***<br>Location of locoregional metastasis/metastases	☐ Vessel(s)<br>☐ Nerve(s)<br>☐ Lymph node(s)<br>☐ Surrounding tissue<br>☐ Other
4.5.1.1.1	***If 'Location of locoregional metastasis/metastases' is equal to 'Other' answer this question:***<br>Other locoregional location	
4.5.1.2	***If 'Location of metastasis/metastases' is equal to 'Distant' answer this question:***<br>Location of distant metastasis/metastases	☐ Bone<br>☐ Liver<br>☐ Lung<br>☐ Brain<br>☐ Adrenal gland<br>☐ Peritoneum<br>☐ Lymph node<br>☐ Skin<br>☐ Other

4.5.1.2.1	***If 'Location of distant metastasis/metastases' is equal to 'Other' answer this question:***<br>Other distant location	
4.5.2	***If 'Malignant paraganglioma upon first visit?' is equal to 'Yes' answer this question:***<br>Did paraganglioma/pheo become malignant over time?	◯ Yes<br>◯ No<br>◯ Unknown
4.5.2.1	***If 'Did paraganglioma/pheo become malignant over time?' is equal to 'Yes' answer this question:***<br>Time between initial diagnosis and malignant disease in months	month(s)

5. Clinical and laboratory - Urine sample

Number	Question	Answers
	NOTE: please fill in the results of the first obtained urine sample	
5.1	Urine sample obtained?	◯ Yes<br>◯ No
	If specific hormone is not measured, please fill in: 0	
5.1.2	***If 'Urine sample obtained?' is equal to 'Yes' answer this question:***<br>Date of first urine sample<br>*Warning shown if field's value is larger than NOW: 'Date of sample cannot be in the future'*	☐ ☐ ☐ *(dd-mm-yyyy)*
5.1.3	***If 'Urine sample obtained?' is equal to 'Yes' answer this question:***<br>Vanillylmandelic acid (VMA) / 24h	☐ umol/24h
5.1.4	***If 'Urine sample obtained?' is equal to 'Yes' answer this question:***<br>Metanephrine / 24h	☐ umol/24h
5.1.5	***If 'Urine sample obtained?' is equal to 'Yes' answer this question:***<br>Normetanephrine / 24h	☐ umol/24h
5.1.6	***If 'Urine sample obtained?' is equal to 'Yes' answer this question:***<br>Free adrenaline (epinephrine) / 24h	☐ nmol/24h
5.1.7	***If 'Urine sample obtained?' is equal to 'Yes' answer this question:***<br>Free noradrenaline (norepinephrine) / 24h	☐ nmol/24h
5.1.8	***If 'Urine sample obtained?' is equal to 'Yes' answer this question:***<br>Free dopamine / 24h	☐ nmol/24h
5.1.9	***If 'Urine sample obtained?' is equal to 'Yes' answer this question:***<br>Positive urine test?	

5.1.9.1	***If 'Positive urine test?' is equal to 'Yes' answer this question:***<br>Experiencing symptoms?	☐ Headache<br>☐ Excessive sweating<br>☐ Paleness<br>☐ Anxiety<br>☐ Tremor<br>☐ Shortness of breath<br>☐ High blood pressure<br>☐ Rapid heart beat<br>☐ Exhaustion/tiredness<br>☐ Rushed feeling<br>☐ Other<br>☐ None<br>☐ Unknown
5.1.9.1.1	***If 'Experiencing symptoms?' is equal to 'Other' answer this question:***<br>What other symptoms?	
5.1.9.2	***If 'Positive urine test?' is equal to 'Yes' answer this question:***<br>Medication obtained for symptoms?	○ Yes<br>○ No<br>○ Unknown
5.1.9.2.1	***If 'Medication obtained for symptoms?' is equal to 'Yes' answer this question:***<br>What medication?	
5.1.9.2.2	***If 'Medication obtained for symptoms?' is equal to 'Yes' answer this question:***<br>Normalisation of hormones from obtained treatment?	○ Yes<br>○ No<br>○ Unknown<br>○ No treatment obtained

6. Clinical and laboratory - Blood test

Number	Question	Answers
	NOTE: please fill in the FIRST blood results	
6.1	Blood test obtained?	◯ Yes<br>◯ No
6.1.1	***If 'Blood test obtained?' is equal to 'Yes' answer this question:***<br>Date of first blood test<br>*Warning shown if field's value is larger than NOW: 'Date cannot be in the future'*	[ ] [ ] [ ] *(dd-mm-yyyy)*
6.1.2	***If 'Blood test obtained?' is equal to 'Yes' answer this question:***<br>Measurement position	◯ Lying down<br>◯ Sitting<br>◯ Standing<br>◯ Unknown
6.1.3	***If 'Blood test obtained?' is equal to 'Yes' answer this question:***<br>Does the patient use any interfering medication during blood withdrawel?	◯ Yes<br>◯ No<br>◯ Unknown
6.1.3.1	***If 'Does the patient use any interfering medication during blood withdrawel?' is equal to 'Yes' answer this question:***<br>What medication does the patient use?	☐ SSRI's (Selective Serotonine Reuptake Inhibitor)<br>☐ TCA (Tricyclic antidepressant)<br>☐ MAO inhibitor (Monoamine Oxidase)<br>☐ Ethanol<br>☐ Other
6.1.3.1.1	***If 'What medication does the patient use?' is equal to 'Other' answer this question:***<br>Other medication	[ ]
6.1.4	***If 'Blood test obtained?' is equal to 'Yes' answer this question:***<br>Free 3-methoxytyramine	[ ] nmol/L
6.1.5	***If 'Blood test obtained?' is equal to 'Yes' answer this question:***<br>Free metanephrine	[ ] nmol/L
6.1.6	***If 'Blood test obtained?' is equal to 'Yes' answer this question:***<br>Free normetanephrine	[ ] nmol/L

6.1.7	***If 'Blood test obtained?' is equal to 'Yes' answer this question:***<br>Positive blood screening	
6.1.7.1	***If 'Positive blood screening' is equal to 'Yes' answer this question:***<br>Blood sample drawn under ideal conditions?	○ Yes<br>○ No<br>○ Unknown
6.1.7.1.1	***If 'Blood sample drawn under ideal conditions?' is equal to 'Yes' answer this question:***<br>Normalisation of hormones after blood drawn under ideal conditions?	○ Yes<br>○ No<br>○ Unknown
6.1.7.2	***If 'Positive blood screening' is equal to 'Yes' answer this question:***<br>Experiencing symptoms?	☐ Headache<br>☐ Excessive sweating<br>☐ Paleness<br>☐ Anxiety<br>☐ Tremor<br>☐ Shortness of breath<br>☐ High blood pressure<br>☐ Rapid heart beat<br>☐ Exhaustion/tiredness<br>☐ Rushed feeling<br>☐ Other<br>☐ None<br>☐ Unknown
6.1.7.2.1	***If 'Experiencing symptoms?' is equal to 'High blood pressure' answer this question:***<br>Systolic blood pressure	______ mmHg
6.1.7.2.2	***If 'Experiencing symptoms?' is equal to 'High blood pressure' answer this question:***<br>Diastolic blood pressure	______ mmHg
6.1.7.2.3	***If 'Experiencing symptoms?' is equal to 'Rapid heart beat' answer this question:***<br>Heart rate	______ beats/min
6.1.7.2.4	***If 'Experiencing symptoms?' is equal to 'Other' answer this question:***<br>What other symptoms?	
6.1.7.3	***If 'Positive blood screening' is equal to 'Yes' answer this question:***<br>Medicinal treatment obtained for symptoms?	○ Yes<br>○ No<br>○ Unknown

6.1.7.3.1	***If 'Medicinal treatment obtained for symptoms?' is equal to 'Yes' answer this question:*** What type of medicinal treatment?	
6.1.7.3.2	***If 'Medicinal treatment obtained for symptoms?' is equal to 'Yes' answer this question:*** Normalisation of hormones due to obtained treatment?	◯ Yes<br>◯ No<br>◯ Unknown<br>◯ No treatment obtained

7. Clinical and laboratory - Imaging techniques

Number	Question	Answers
	NOTE: please only fill in results of the scans UPON diagnosis. Later there will be room to fill in additional scans showing tumor changes.	
7.1	Imaging techniques upon diagnosis	☐ Ultrasound (US) of the neck<br>☐ US of the neck with biopsy<br>☐ CT of the head/neck<br>☐ CT-angiography<br>☐ CT-thorax<br>☐ CT-abdomen<br>☐ MRI of the head/neck<br>☐ MRI paraganglioma (whole body)<br>☐ FDG-PET<br>☐ MIBG<br>☐ F-DOPA<br>☐ 68Ga-DOTA scan<br>☐ Other<br>☐ None<br>☐ Other hospital
7.1.1	***If 'Imaging techniques upon diagnosis' is equal to 'Ultrasound (US) of the neck' answer this question:***<br>Date of the US	____ ____ ______ *(dd-mm-yyyy)*
7.1.2	***If 'Imaging techniques upon diagnosis' is equal to 'Ultrasound (US) of the neck' answer this question:***<br>Abnormalities on ultrasound?	○ Yes<br>○ No
7.1.2.1	***If 'Abnormalities on ultrasound?' is equal to 'Yes' answer this question:***<br>Detected paragangliomas	☐ Carotid body left<br>☐ Carotid body right<br>☐ Tympanic left<br>☐ Tympanic right<br>☐ Jugular left<br>☐ Jugular right<br>☐ Vagal left<br>☐ Vagal right<br>☐ Thoracic<br>☐ Abdominal<br>☐ Pheochromocytoma<br>☐ Other<br>☐ None<br>☐ Unknown

7.1.2.1.1	***If 'Detected paragangliomas' is equal to 'Thoracic' answer this question:***<br>Specification of thoracic paraganglioma	☐ Pulmonary<br>☐ Mediastinal<br>☐ Diaphragmatic<br>☐ Spine<br>☐ Para aortal<br>☐ Cardiac<br>☐ Aorticopulmonary<br>☐ Para tracheal<br>☐ Other
7.1.2.1.2	***If 'Detected paragangliomas' is equal to 'Abdominal' answer this question:***<br>Specification of abdominal paraganglioma	☐ Mesenteric<br>☐ Para aortal<br>☐ Organ of Zuckerkandl<br>☐ Duodenal/ ampulla of Vater<br>☐ Bladder<br>☐ Colorectal<br>☐ Retroperitoneal<br>☐ Gonadal<br>☐ Peri-renal<br>☐ Sympathetic trunc<br>☐ Other
7.1.2.1.3	***If 'Detected paragangliomas' is equal to 'Pheochromocytoma' answer this question:***<br>Location of pheochromocytoma	☐ Left adrenal gland<br>☐ Right adrenal gland<br>☐ Other
7.1.2.1.4	***If 'Detected paragangliomas' is equal to 'Other' answer this question:***<br>Location of other paraganglioma	[ ]
7.1.3	***If 'Imaging techniques upon diagnosis' is equal to 'US of the neck with biopsy' answer this question:***<br>Date of US + biopsie	[ ] [ ] [ ] *(dd-mm-yyyy)*
7.1.4	***If 'Imaging techniques upon diagnosis' is equal to 'US of the neck with biopsy' answer this question:***<br>Result of biopsy	◯ Paraganglioma<br>◯ Normal tissue from biopsy place<br>◯ Other malignancy
7.1.5	***If 'Imaging techniques upon diagnosis' is equal to 'US of the neck with biopsy' answer this question:***<br>PA obtained?	◯ Yes<br>◯ No
7.1.5.1	***If 'PA obtained?' is equal to 'Yes' answer this question:***<br>PA number of biopsy	[ ]
7.1.6	***If 'Imaging techniques upon diagnosis' is equal to 'US of the neck with biopsy' answer this question:***<br>Abnormalities on ultrasound?	◯ Yes<br>◯ No

7.1.6.1	***If 'Abnormalities on ultrasound?' is equal to 'Yes' answer this question:***<br>Detected paragangliomas	☐ Carotid body left<br>☐ Carotid body right<br>☐ Tympanic left<br>☐ Tympanic right<br>☐ Jugular left<br>☐ Jugular right<br>☐ Vagal left<br>☐ Vagal right<br>☐ Thoracic<br>☐ Abdominal<br>☐ Pheochromocytoma<br>☐ Other<br>☐ None<br>☐ Unknown
7.1.6.1.1	***If 'Detected paragangliomas' is equal to 'Thoracic' answer this question:***<br>Specification of thoracic paraganglioma	☐ Pulmonary<br>☐ Mediastinal<br>☐ Diaphragmatic<br>☐ Spine<br>☐ Para aortal<br>☐ Cardiac<br>☐ Aorticopulmonary<br>☐ Para tracheal<br>☐ Other
7.1.6.1.2	***If 'Detected paragangliomas' is equal to 'Abdominal' answer this question:***<br>Specification of abdominal paraganglioma	☐ Mesenteric<br>☐ Para aortal<br>☐ Organ of Zuckerkandl<br>☐ Duodenal/ ampulla of Vater<br>☐ Bladder<br>☐ Colorectal<br>☐ Retroperitoneal<br>☐ Gonadal<br>☐ Peri-renal<br>☐ Sympathetic trunc<br>☐ Other
7.1.6.1.3	***If 'Detected paragangliomas' is equal to 'Pheochromocytoma' answer this question:***<br>Location of pheochromocytoma	☐ Left adrenal gland<br>☐ Right adrenal gland<br>☐ Other
7.1.6.1.4	***If 'Detected paragangliomas' is equal to 'Other' answer this question:***<br>Location of other paraganglioma	

7.1.7	***If 'Imaging techniques upon diagnosis' is equal to 'CT of the head/neck' answer this question:***<br>Date of CT of the head & neck	☐ ☐ ☐ *(dd-mm-yyyy)*
7.1.8	***If 'Imaging techniques upon diagnosis' is equal to 'CT of the head/neck' answer this question:***<br>Abnormalities on CT of the head & neck?	○ Yes<br>○ No
7.1.8.1	***If 'Abnormalities on CT of the head & neck?' is equal to 'Yes' answer this question:***<br>Detected paragangliomas	☐ Carotid body left<br>☐ Carotid body right<br>☐ Tympanic left<br>☐ Typmanic right<br>☐ Jugular left<br>☐ Jugular right<br>☐ Vagal left<br>☐ Vagal right<br>☐ Other<br>☐ Unknown
7.1.9	***If 'Imaging techniques upon diagnosis' is equal to 'CT-angiography' answer this question:***<br>Date of CT angiography	☐ ☐ ☐ *(dd-mm-yyyy)*
7.1.10	***If 'Imaging techniques upon diagnosis' is equal to 'CT-angiography' answer this question:***<br>Abnormalities on angiography?	○ Yes<br>○ No
7.1.10.1	***If 'Abnormalities on angiography?' is equal to 'Yes' answer this question:***<br>Detected paragangliomas	☐ Carotid body left<br>☐ Carotid body right<br>☐ Tympanic left<br>☐ Tympanic right<br>☐ Jugular left<br>☐ Jugular right<br>☐ Vagal left<br>☐ Vagal right<br>☐ Thoracic<br>☐ Abdominal<br>☐ Pheochromocytoma<br>☐ Other<br>☐ None<br>☐ Unknown

7.1.10.1.1	***If 'Detected paragangliomas' is equal to 'Thoracic' answer this question:***<br>Specification of thoracic paraganglioma	☐ Pulmonary<br>☐ Mediastinal<br>☐ Diaphragmatic<br>☐ Spine<br>☐ Para aortal<br>☐ Cardiac<br>☐ Aorticopulmonary<br>☐ Para tracheal<br>☐ Other
7.1.10.1.2	***If 'Detected paragangliomas' is equal to 'Abdominal' answer this question:***<br>Specification of abdominal paraganglioma	☐ Mesenteric<br>☐ Para aortal<br>☐ Organ of Zuckerkandl<br>☐ Duodenal/ ampulla of Vater<br>☐ Bladder<br>☐ Colorectal<br>☐ Retroperitoneal<br>☐ Gonadal<br>☐ Peri-renal<br>☐ Sympathetic trunc<br>☐ Other
7.1.10.1.3	***If 'Detected paragangliomas' is equal to 'Pheochromocytoma' answer this question:***<br>Location of pheochromocytoma	☐ Left adrenal gland<br>☐ Right adrenal gland<br>☐ Other
7.1.10.1.4	***If 'Detected paragangliomas' is equal to 'Other' answer this question:***<br>Location of other paraganglioma	________
7.1.11	***If 'Imaging techniques upon diagnosis' is equal to 'CT-abdomen' answer this question:***<br>Date of CT abdomen	___ ___ ____ *(dd-mm-yyyy)*
7.1.12	***If 'Imaging techniques upon diagnosis' is equal to 'CT-abdomen' answer this question:***<br>Abnormalities on CT abdomen?	○ Yes<br>○ No

7.1.12.1	***If 'Abnormalities on CT abdomen?' is equal to 'Yes' answer this question:***<br>Detected paragangliomas	☐ Carotid body left<br>☐ Carotid body right<br>☐ Tympanic left<br>☐ Tympanic right<br>☐ Jugular left<br>☐ Jugular right<br>☐ Vagal left<br>☐ Vagal right<br>☐ Thoracic<br>☐ Abdominal<br>☐ Pheochromocytoma<br>☐ Other<br>☐ None<br>☐ Unknown
7.1.12.1.1	***If 'Detected paragangliomas' is equal to 'Abdominal' answer this question:***<br>Specification of abdominal paraganglioma	☐ Mesenteric<br>☐ Para aortal<br>☐ Organ of Zuckerkandl<br>☐ Duodenal/ ampulla of Vater<br>☐ Bladder<br>☐ Colorectal<br>☐ Retroperitoneal<br>☐ Gonadal<br>☐ Peri-renal<br>☐ Sympathetic trunc<br>☐ Other
7.1.13	***If 'Imaging techniques upon diagnosis' is equal to 'CT-thorax' answer this question:***<br>Date of CT-thorax	___ ___ ___ *(dd-mm-yyyy)*
7.1.14	***If 'Imaging techniques upon diagnosis' is equal to 'CT-thorax' answer this question:***<br>Abnormalities on CT thorax?	○ Yes<br>○ No

7.1.14.1	***If 'Abnormalities on CT thorax?' is equal to 'Yes' answer this question:***<br>Detected paragangliomas	☐ Carotid body left<br>☐ Carotid body right<br>☐ Tympanic left<br>☐ Tympanic right<br>☐ Jugular left<br>☐ Jugular right<br>☐ Vagal left<br>☐ Vagal right<br>☐ Thoracic<br>☐ Abdominal<br>☐ Pheochromocytoma<br>☐ Other<br>☐ None<br>☐ Unknown
7.1.14.1.1	***If 'Detected paragangliomas' is equal to 'Thoracic' answer this question:***<br>Specification of thoracic paraganglioma	☐ Pulmonary<br>☐ Mediastinal<br>☐ Diaphragmatic<br>☐ Spine<br>☐ Para aortal<br>☐ Cardiac<br>☐ Aorticopulmonary<br>☐ Para tracheal<br>☐ Other
7.1.15	***If 'Imaging techniques upon diagnosis' is equal to 'MRI of the head/neck' answer this question:***<br>Date of head/neck MRI	[ ] [ ] [ ] *(dd-mm-yyyy)*
7.1.16	***If 'Imaging techniques upon diagnosis' is equal to 'MRI of the head/neck' answer this question:***<br>Abnormalities on head/neck MRI?	○ Yes<br>○ No
7.1.16.1	***If 'Abnormalities on head/neck MRI?' is equal to 'Yes' answer this question:***<br>Detected paragangliomas	☐ Carotid body left<br>☐ Carotid body right<br>☐ Tympanic left<br>☐ Typmanic right<br>☐ Jugular left<br>☐ Jugular right<br>☐ Vagal left<br>☐ Vagal right<br>☐ Other<br>☐ Unknown
7.1.17	***If 'Imaging techniques upon diagnosis' is equal to 'MRI paraganglioma (whole body)' answer this question:***<br>Date of MRI paraganglioma (whole body)	[ ] [ ] [ ] *(dd-mm-yyyy)*

7.1.18	***If 'Imaging techniques upon diagnosis' is equal to 'MRI paraganglioma (whole body)' answer this question:*** Abnormalities on MRI paraganglioma (whole body)?	○ Yes<br>○ No
7.1.18.1	***If 'Abnormalities on MRI paraganglioma (whole body)?' is equal to 'Yes' answer this question:*** Detected paragangliomas	☐ Carotid body left<br>☐ Carotid body right<br>☐ Tympanic left<br>☐ Tympanic right<br>☐ Jugular left<br>☐ Jugular right<br>☐ Vagal left<br>☐ Vagal right<br>☐ Thoracic<br>☐ Abdominal<br>☐ Pheochromocytoma<br>☐ Other<br>☐ None<br>☐ Unknown
7.1.18.1.1	***If 'Detected paragangliomas' is equal to 'Thoracic' answer this question:*** Specification of thoracic paraganglioma	☐ Pulmonary<br>☐ Mediastinal<br>☐ Diaphragmatic<br>☐ Spine<br>☐ Para aortal<br>☐ Cardiac<br>☐ Aorticopulmonary<br>☐ Para tracheal<br>☐ Other
7.1.18.1.2	***If 'Detected paragangliomas' is equal to 'Abdominal' answer this question:*** Specification of abdominal paraganglioma	☐ Mesenteric<br>☐ Para aortal<br>☐ Organ of Zuckerkandl<br>☐ Duodenal/ ampulla of Vater<br>☐ Bladder<br>☐ Colorectal<br>☐ Retroperitoneal<br>☐ Gonadal<br>☐ Peri-renal<br>☐ Sympathetic trunc<br>☐ Other
7.1.18.1.3	***If 'Detected paragangliomas' is equal to 'Pheochromocytoma' answer this question:*** Location of pheochromocytoma	☐ Left adrenal gland<br>☐ Right adrenal gland<br>☐ Other

7.1.18.1.4	***If 'Detected paragangliomas' is equal to 'Other' answer this question:***<br>Location of other paraganglioma	
7.1.19	***If 'Imaging techniques upon diagnosis' is equal to 'FDG-PET' answer this question:***<br>Date of FDG-PET	*(dd-mm-yyyy)*
7.1.20	***If 'Imaging techniques upon diagnosis' is equal to 'FDG-PET' answer this question:***<br>Abnormalities on FDG-PET?	○ Yes<br>○ No
7.1.20.1	***If 'Abnormalities on FDG-PET?' is equal to 'Yes' answer this question:***<br>Detected paragangliomas	☐ Carotid body left<br>☐ Carotid body right<br>☐ Tympanic left<br>☐ Tympanic right<br>☐ Jugular left<br>☐ Jugular right<br>☐ Vagal left<br>☐ Vagal right<br>☐ Thoracic<br>☐ Abdominal<br>☐ Pheochromocytoma<br>☐ Other<br>☐ None<br>☐ Unknown
7.1.20.1.1	***If 'Detected paragangliomas' is equal to 'Thoracic' answer this question:***<br>Specification of thoracic paraganglioma	☐ Pulmonary<br>☐ Mediastinal<br>☐ Diaphragmatic<br>☐ Spine<br>☐ Para aortal<br>☐ Cardiac<br>☐ Aorticopulmonary<br>☐ Para tracheal<br>☐ Other
7.1.20.1.2	***If 'Detected paragangliomas' is equal to 'Abdominal' answer this question:***<br>Specification of abdominal paraganglioma	☐ Mesenteric<br>☐ Para aortal<br>☐ Organ of Zuckerkandl<br>☐ Duodenal/ ampulla of Vater<br>☐ Bladder<br>☐ Colorectal<br>☐ Retroperitoneal<br>☐ Gonadal<br>☐ Peri-renal<br>☐ Sympathetic trunc<br>☐ Other

7.1.20.1.3	***If 'Detected paragangliomas' is equal to 'Pheochromocytoma' answer this question:***<br>Location of pheochromocytoma	☐ Left adrenal gland<br>☐ Right adrenal gland<br>☐ Other
7.1.20.1.4	***If 'Detected paragangliomas' is equal to 'Other' answer this question:***<br>Location of other paraganglioma	
7.1.21	***If 'Imaging techniques upon diagnosis' is equal to 'MIBG' answer this question:***<br>Date of MIBG	*(dd-mm-yyyy)*
7.1.22	***If 'Imaging techniques upon diagnosis' is equal to 'MIBG' answer this question:***<br>Abnormalities on MIBG?	○ Yes<br>○ No
7.1.22.1	***If 'Abnormalities on MIBG?' is equal to 'Yes' answer this question:***<br>Detected paragangliomas	☐ Carotid body left<br>☐ Carotid body right<br>☐ Tympanic left<br>☐ Tympanic right<br>☐ Jugular left<br>☐ Jugular right<br>☐ Vagal left<br>☐ Vagal right<br>☐ Thoracic<br>☐ Abdominal<br>☐ Pheochromocytoma<br>☐ Other<br>☐ None<br>☐ Unknown
7.1.22.1.1	***If 'Detected paragangliomas' is equal to 'Thoracic' answer this question:***<br>Specification of thoracic paraganglioma	☐ Pulmonary<br>☐ Mediastinal<br>☐ Diaphragmatic<br>☐ Spine<br>☐ Para aortal<br>☐ Cardiac<br>☐ Aorticopulmonary<br>☐ Para tracheal<br>☐ Other

7.1.22.1.2	***If 'Detected paragangliomas' is equal to 'Abdominal' answer this question:***<br>Specification of abdominal paraganglioma	☐ Mesenteric<br>☐ Para aortal<br>☐ Organ of Zuckerkandl<br>☐ Duodenal/ ampulla of Vater<br>☐ Bladder<br>☐ Colorectal<br>☐ Retroperitoneal<br>☐ Gonadal<br>☐ Peri-renal<br>☐ Sympathetic trunc<br>☐ Other
7.1.22.1.3	***If 'Detected paragangliomas' is equal to 'Pheochromocytoma' answer this question:***<br>Location of pheochromocytoma	☐ Left adrenal gland<br>☐ Right adrenal gland<br>☐ Other
7.1.22.1.4	***If 'Detected paragangliomas' is equal to 'Other' answer this question:***<br>Location of other paraganglioma	
7.1.23	***If 'Imaging techniques upon diagnosis' is equal to 'F-DOPA' answer this question:***<br>Date of F-DOPA	*(dd-mm-yyyy)*
7.1.24	***If 'Imaging techniques upon diagnosis' is equal to 'F-DOPA' answer this question:***<br>Abnormalities on F-DOPA?	○ Yes<br>○ No
7.1.24.1	***If 'Abnormalities on F-DOPA?' is equal to 'Yes' answer this question:***<br>Detected paragangliomas	☐ Carotid body left<br>☐ Carotid body right<br>☐ Tympanic left<br>☐ Tympanic right<br>☐ Jugular left<br>☐ Jugular right<br>☐ Vagal left<br>☐ Vagal right<br>☐ Thoracic<br>☐ Abdominal<br>☐ Pheochromocytoma<br>☐ Other<br>☐ None<br>☐ Unknown

7.1.24.1.1	***If 'Detected paragangliomas' is equal to 'Thoracic' answer this question:***<br>Specification of thoracic paraganglioma	☐ Pulmonary<br>☐ Mediastinal<br>☐ Diaphragmatic<br>☐ Spine<br>☐ Para aortal<br>☐ Cardiac<br>☐ Aorticopulmonary<br>☐ Para tracheal<br>☐ Other
7.1.24.1.2	***If 'Detected paragangliomas' is equal to 'Abdominal' answer this question:***<br>Specification of abdominal paraganglioma	☐ Mesenteric<br>☐ Para aortal<br>☐ Organ of Zuckerkandl<br>☐ Duodenal/ ampulla of Vater<br>☐ Bladder<br>☐ Colorectal<br>☐ Retroperitoneal<br>☐ Gonadal<br>☐ Peri-renal<br>☐ Sympathetic trunc<br>☐ Other
7.1.24.1.3	***If 'Detected paragangliomas' is equal to 'Pheochromocytoma' answer this question:***<br>Location of pheochromocytoma	☐ Left adrenal gland<br>☐ Right adrenal gland<br>☐ Other
7.1.24.1.4	***If 'Detected paragangliomas' is equal to 'Other' answer this question:***<br>Location of other paraganglioma	
7.1.25	***If 'Imaging techniques upon diagnosis' is equal to '68Ga-DOTA scan' answer this question:***<br>Date of 68Ga-DOTA scan	*(dd-mm-yyyy)*
7.1.26	***If 'Imaging techniques upon diagnosis' is equal to '68Ga-DOTA scan' answer this question:***<br>Specific DOTA scan type	◯ DOTATOC<br>◯ DOTATATE<br>◯ DOTANOC<br>◯ Unknown
7.1.27	***If 'Imaging techniques upon diagnosis' is equal to '68Ga-DOTA scan' answer this question:***<br>Abnormalities on 68Ga-DOTA?	◯ Yes<br>◯ No

7.1.27.1	***If 'Abnormalities on 68Ga-DOTA?' is equal to 'Yes' answer this question:***<br>Detected paragangliomas	☐ Carotid body left<br>☐ Carotid body right<br>☐ Tympanic left<br>☐ Tympanic right<br>☐ Jugular left<br>☐ Jugular right<br>☐ Vagal left<br>☐ Vagal right<br>☐ Thoracic<br>☐ Abdominal<br>☐ Pheochromocytoma<br>☐ Other<br>☐ None<br>☐ Unknown
7.1.27.1.1	***If 'Detected paragangliomas' is equal to 'Thoracic' answer this question:***<br>Specification of thoracic paraganglioma	☐ Pulmonary<br>☐ Mediastinal<br>☐ Diaphragmatic<br>☐ Spine<br>☐ Para aortal<br>☐ Cardiac<br>☐ Aorticopulmonary<br>☐ Para tracheal<br>☐ Other
7.1.27.1.2	***If 'Detected paragangliomas' is equal to 'Abdominal' answer this question:***<br>Specification of abdominal paraganglioma	☐ Mesenteric<br>☐ Para aortal<br>☐ Organ of Zuckerkandl<br>☐ Duodenal/ ampulla of Vater<br>☐ Bladder<br>☐ Colorectal<br>☐ Retroperitoneal<br>☐ Gonadal<br>☐ Peri-renal<br>☐ Sympathetic trunc<br>☐ Other
7.1.27.1.3	***If 'Detected paragangliomas' is equal to 'Pheochromocytoma' answer this question:***<br>Location of pheochromocytoma	☐ Left adrenal gland<br>☐ Right adrenal gland<br>☐ Other
7.1.27.1.4	***If 'Detected paragangliomas' is equal to 'Other' answer this question:***<br>Location of other paraganglioma	

7.1.28	***If 'Imaging techniques upon diagnosis' is equal to 'Other' answer this question:***<br>Date of other scan	☐ ☐ ☐ *(dd-mm-yyyy)*
7.1.29	***If 'Imaging techniques upon diagnosis' is equal to 'Other' answer this question:***<br>Type of other scan	☐
7.1.30	***If 'Imaging techniques upon diagnosis' is equal to 'Other' answer this question:***<br>Abnormalities on other scan?	○ Yes<br>○ No
7.1.30.1	***If 'Abnormalities on other scan?' is equal to 'Yes' answer this question:***<br>Detected paragangliomas	☐ Carotid body left<br>☐ Carotid body right<br>☐ Tympanic left<br>☐ Tympanic right<br>☐ Jugular left<br>☐ Jugular right<br>☐ Vagal left<br>☐ Vagal right<br>☐ Thoracic<br>☐ Abdominal<br>☐ Pheochromocytoma<br>☐ Other<br>☐ None<br>☐ Unknown
7.1.30.1.1	***If 'Detected paragangliomas' is equal to 'Thoracic' answer this question:***<br>Specification of thoracic paraganglioma	☐ Pulmonary<br>☐ Mediastinal<br>☐ Diaphragmatic<br>☐ Spine<br>☐ Para aortal<br>☐ Cardiac<br>☐ Aorticopulmonary<br>☐ Para tracheal<br>☐ Other

7.1.30.1.2	***If 'Detected paragangliomas' is equal to 'Abdominal' answer this question:***<br>Specification of abdominal paraganglioma	☐ Mesenteric<br>☐ Para aortal<br>☐ Organ of Zuckerkandl<br>☐ Duodenal/ ampulla of Vater<br>☐ Bladder<br>☐ Colorectal<br>☐ Retroperitoneal<br>☐ Gonadal<br>☐ Peri-renal<br>☐ Sympathetic trunc<br>☐ Other
7.1.30.1.3	***If 'Detected paragangliomas' is equal to 'Pheochromocytoma' answer this question:***<br>Location of pheochromocytoma	☐ Left adrenal gland<br>☐ Right adrenal gland<br>☐ Other
7.1.30.1.4	***If 'Detected paragangliomas' is equal to 'Other' answer this question:***<br>Other result	

8. Diagnosis - Diagnosis

Number	Question	Answers
	The following questions focus on all (previously) diagnosed paraganglioma.	
8.1	***If 'Does the patient have a known genetic mutation' is equal to 'Yes' answer this question:***<br>Diagnosed mutation	
8.2	***If 'Does the patient have a known genetic mutation' is equal to 'Yes' answer this question:***<br>Classification of mutation	◯ Pathogenic<br>◯ Likely pathogenic<br>◯ Variant of uncertain significance (VUS)<br>◯ Likely benign<br>◯ Benign
8.3	***If 'Does the patient have a known genetic mutation' is equal to 'Yes' answer this question:***<br>Genotype of mutation	
8.4	All diagnosed paraganglioma	☐ Carotid body left<br>☐ Carotid body right<br>☐ Tympanic left<br>☐ Tympanic right<br>☐ Jugular left<br>☐ Jugular right<br>☐ Vagal left<br>☐ Vagal right<br>☐ Thoracic<br>☐ Abdominal<br>☐ Pheochromocytoma<br>☐ Other<br>☐ None<br>☐ Unknown
8.4.1	***If 'All diagnosed paraganglioma' is equal to 'Abdominal' answer this question:***<br>Location of abdominal PGL	☐ Mesenteric<br>☐ Para aortal<br>☐ Organ of Zuckerkandl<br>☐ Duodenal/ ampulla of Vater<br>☐ Bladder<br>☐ Colorectal<br>☐ Retroperitoneal<br>☐ Gonadal<br>☐ Peri-renal<br>☐ Sympathetic trunc<br>☐ Other

8.4.1.1	***If 'Location of abdominal PGL' is equal to 'Other' answer this question:*** Other location of abdominal PGL	
8.4.2	***If 'All diagnosed paraganglioma' is equal to 'Abdominal' answer this question:*** Most recent size of abdominal paraganglioma in mm	mm
8.4.3	***If 'All diagnosed paraganglioma' is equal to 'Abdominal' answer this question:*** Year of abdominal paraganglioma diagnosis	*(yyyy)*
8.4.4	***If 'All diagnosed paraganglioma' is equal to 'Thoracic' answer this question:*** Location of thoracic PGL	☐ Pulmonary<br>☐ Mediastinal<br>☐ Diaphragmatic<br>☐ Spine<br>☐ Para aortal<br>☐ Cardiac<br>☐ Aorticopulmonary<br>☐ Para tracheal<br>☐ Other
8.4.4.1	***If 'Location of thoracic PGL' is equal to 'Para tracheal' answer this question:*** Other location of thoracic PGL	
8.4.5	***If 'All diagnosed paraganglioma' is equal to 'Thoracic' answer this question:*** Most recent size of thoracic paraganglioma in mm	mm
8.4.6	***If 'All diagnosed paraganglioma' is equal to 'Thoracic' answer this question:*** Year of thoracic paraganglioma diagnosis	*(yyyy)*
8.4.7	***If 'All diagnosed paraganglioma' is equal to 'Other' answer this question:*** Location of other paraganglioma	
8.4.8	***If 'All diagnosed paraganglioma' is equal to 'Other' answer this question:*** Most recent size of other paraganglioma in mm	mm
8.4.9	***If 'All diagnosed paraganglioma' is equal to 'Other' answer this question:*** Year of 'other paraganglioma' diagnosis	*(yyyy)*
8.4.10	***If 'All diagnosed paraganglioma' is equal to 'Carotid body left' answer this question:*** Classification of left carotid body paraganglioma (CB PGL)	◯ Shamblin 1<br>◯ Shamblin 2<br>◯ Shamblin 3<br>◯ Unknown

8.4.11	***If 'All diagnosed paraganglioma' is equal to 'Carotid body left' answer this question:***<br>Most recent size of left carotid body paraganglioma in mm	[ ] mm
8.4.12	***If 'All diagnosed paraganglioma' is equal to 'Carotid body left' answer this question:***<br>Year of left carotid body paraganglioma diagnosis	[ ] *(yyyy)*
8.4.13	***If 'All diagnosed paraganglioma' is equal to 'Carotid body right' answer this question:***<br>Classification of right carotid body paraganglioma (CB PGL)	○ Shamblin 1<br>○ Shamblin 2<br>○ Shamblin 3<br>○ Unknown
8.4.14	***If 'All diagnosed paraganglioma' is equal to 'Carotid body right' answer this question:***<br>Most recent size of right carotid body paraganglioma in mm	[ ] mm
8.4.15	***If 'All diagnosed paraganglioma' is equal to 'Carotid body right' answer this question:***<br>Year of right carotid body paraganglioma diagnosis	[ ] *(yyyy)*
8.4.16	***If 'All diagnosed paraganglioma' is equal to 'Tympanic left' answer this question:***<br>Classification of left tympanic paraganglioma (T PGL)	○ Type A (tympanicum)<br>○ Type B (hypotympanicum)<br>○ Type C<br>○ Type C1<br>○ Type C2<br>○ Type C3<br>○ Type D<br>○ Type D1<br>○ Type D2<br>○ Unknown
8.4.17	***If 'All diagnosed paraganglioma' is equal to 'Tympanic left' answer this question:***<br>Most recent size of left tympanic paraganglioma in mm	[ ] mm
8.4.18	***If 'All diagnosed paraganglioma' is equal to 'Tympanic left' answer this question:***<br>Year of left tympanic diagnosis	[ ] *(yyyy)*
8.4.19	***If 'All diagnosed paraganglioma' is equal to 'Tympanic right' answer this question:***<br>Classification of right tympanic paraganglioma (T PGL)	○ Type A (tympanicum)<br>○ Type B (hypotympanicum)<br>○ Type C<br>○ Type C1<br>○ Type C2<br>○ Type C3<br>○ Type D<br>○ Type D1<br>○ Type D2<br>○ Unknown

8.4.20	***If 'All diagnosed paraganglioma' is equal to 'Tympanic right' answer this question:***<br>Most recent size of right tympanic paraganglioma in mm	[ ] mm
8.4.21	***If 'All diagnosed paraganglioma' is equal to 'Tympanic right' answer this question:***<br>Year of right tympanic paraganglioma diagnosis	[ ] *(yyyy)*
8.4.22	***If 'All diagnosed paraganglioma' is equal to 'Jugular left' answer this question:***<br>Classification of left jugular paraganglioma (J PGL)	○ Type A (tympanicum)<br>○ Type B (hypotympanicum)<br>○ Type C<br>○ Type C1<br>○ Type C2<br>○ Type C3<br>○ Type D<br>○ Type D1<br>○ Type D2<br>○ Unknown
8.4.23	***If 'All diagnosed paraganglioma' is equal to 'Jugular left' answer this question:***<br>Most recent size of left jugular paraganglioma in mm	[ ] mm
8.4.24	***If 'All diagnosed paraganglioma' is equal to 'Jugular left' answer this question:***<br>Year of left jugular paraganglioma diagnosis	[ ] *(yyyy)*
8.4.25	***If 'All diagnosed paraganglioma' is equal to 'Jugular right' answer this question:***<br>Classification of right jugular paraganglioma (J PGL)	○ Type A (tympanicum)<br>○ Type B (hypotympanicum)<br>○ Type C<br>○ Type C1<br>○ Type C2<br>○ Type C3<br>○ Type D<br>○ Type D1<br>○ Type D2<br>○ Unknown
8.4.26	***If 'All diagnosed paraganglioma' is equal to 'Jugular right' answer this question:***<br>Most recent size of right jugular paraganglioma in mm	[ ] mm
8.4.27	***If 'All diagnosed paraganglioma' is equal to 'Jugular right' answer this question:***<br>Year of right jugular paraganglioma diagnosis	[ ] *(yyyy)*
8.4.28	***If 'All diagnosed paraganglioma' is equal to 'Vagal left' answer this question:***<br>Most recent size of left vagal paraganglioma in mm	[ ] mm
8.4.29	***If 'All diagnosed paraganglioma' is equal to 'Vagal left' answer this question:***<br>Year of left vagal paraganglioma diagnosis	[ ] *(yyyy)*

8.4.30	***If 'All diagnosed paraganglioma' is equal to 'Vagal right' answer this question:***<br>Most recent size of right vagal paraganglioma in mm	mm
8.4.31	***If 'All diagnosed paraganglioma' is equal to 'Vagal right' answer this question:***<br>Year of right vagal paraganglioma diagnosis	*(yyyy)*
8.4.32	***If 'All diagnosed paraganglioma' is equal to 'Pheochromocytoma' answer this question:***<br>Location of pheochromocytoma	○ Left adrenal medulla<br>○ Right adrenal medulla
8.4.33	***If 'All diagnosed paraganglioma' is equal to 'Pheochromocytoma' answer this question:***<br>Most recent size of pheochromocytoma in mm	mm
8.4.34	***If 'All diagnosed paraganglioma' is equal to 'Pheochromocytoma' answer this question:***<br>Year of pheochromocytoma diagnosis	*(yyyy)*
8.4.35	***If 'All diagnosed paraganglioma' is not equal to 'None' answer this question:***<br>Ingrow in surrounding structures?	○ Yes<br>○ No<br>○ Unknown
8.4.35.1	***If 'Ingrow in surrounding structures?' is equal to 'Yes' answer this question:***<br>Ingrow in which structures	☐ a. carotis interna (ACI)<br>☐ a. carotis externa (ACE)<br>☐ a. carotis communis (ACC)<br>☐ n. glossopharyngeus (IX)<br>☐ n. vagus (X)<br>☐ n. accessorius (XI)<br>☐ n. hypoglossus (XII)<br>☐ Skull base<br>☐ v. jugularis<br>☐ Carotis kanaal<br>☐ Vestibulum<br>☐ Labyrinth<br>☐ Cochlea<br>☐ Os petrosum<br>☐ Mastoïd<br>☐ Membrana tympani<br>☐ Other
8.4.35.1.1	***If 'Ingrow in which structures' is equal to 'Other' answer this question:***<br>Other structure	
8.4.36	***If 'All diagnosed paraganglioma' is not equal to 'None' answer this question:***<br>Surgical intervention for any paraganglioma?	○ Yes<br>○ No

8.4.36.1	***If 'Surgical intervention for any paraganglioma?' is equal to 'Yes' answer this question:***<br>How many surgical interventions?	◯ One<br>◯ Multiple
8.4.36.1.1	***If 'How many surgical interventions?' is equal to 'One' answer this question:***<br>Date of surgical intervention	☐ ☐ ☐ *(dd-mm-yyyy)*
8.4.36.1.2	***If 'How many surgical interventions?' is equal to 'One' answer this question:***<br>Type of excision	◯ Primary surgery<br>◯ Tumor recurrence<br>◯ Previous incomplete excision<br>◯ Other
8.4.36.1.2.1	***If 'Type of excision' is equal to 'Other' answer this question:***<br>Reason of other	
8.4.36.1.3	***If 'How many surgical interventions?' is equal to 'One' answer this question:***<br>Which paraganglioma?	☐ Carotid body left<br>☐ Carotid body right<br>☐ Tympanic left<br>☐ Tympanic right<br>☐ Jugular left<br>☐ Jugular right<br>☐ Vagal left<br>☐ Vagal right<br>☐ Thoracic<br>☐ Abdominal<br>☐ Pheochromocytoma<br>☐ Other<br>☐ None<br>☐ Unknown
8.4.36.1.3.1	***If 'Which paraganglioma?' is equal to 'Thoracic' answer this question:***<br>Location of thoracic paraganglioma	◯ Pulmonary<br>◯ Mediastinal<br>◯ Diaphragmatic<br>◯ Spine<br>◯ Para aortal<br>◯ Cardiac<br>◯ Aorticopulmonary<br>◯ Para tracheal<br>◯ Other

8.4.36.1.3.2	***If 'Which paraganglioma?' is equal to 'Abdominal' answer this question:***<br>Location of abdominal paraganglioma	○ Mesenteric<br>○ Para aortal<br>○ Organ of Zuckerkandl<br>○ Duodenal/ ampulla of Vater<br>○ Bladder<br>○ Colorectal<br>○ Retroperitoneal<br>○ Gonadal<br>○ Peri-renal<br>○ Sympathetic trunc<br>○ Other
8.4.36.1.3.3	***If 'Which paraganglioma?' is equal to 'Pheochromocytoma' answer this question:***<br>Location of pheochromocytoma	○ Left adrenal gland<br>○ Right adrenal gland<br>○ Other
8.4.36.1.3.4	***If 'Which paraganglioma?' is equal to 'Other' answer this question:***<br>Location of other paragangllioma	
8.4.36.1.4	***If 'How many surgical interventions?' is equal to 'One' answer this question:***<br>Operation indication	☐ Tumor growth<br>☐ Hormone production<br>☐ Tumor mutation<br>☐ Pressure on surounding structures<br>☐ Malignancy<br>☐ Pregnancy wish<br>☐ Pregnancy<br>☐ Patient's wish<br>☐ Unknown<br>☐ Other
8.4.36.1.4.1	***If 'Operation indication' is equal to 'Other' answer this question:***<br>What other reason?	
8.4.36.1.5	***If 'How many surgical interventions?' is equal to 'One' answer this question:***<br>Preoperative medical preparation	○ Yes<br>○ No<br>○ Unknown
8.4.36.1.5.1	***If 'Preoperative medical preparation' is equal to 'Yes' answer this question:***<br>What medical treatment?	☐ Alpha blockage<br>☐ Beta blockage<br>☐ Other

8.4.36.1.5.2	***If 'Preoperative medical preparation' is equal to 'Yes' answer this question:***<br>Response to medication	☐ Therapy resistant<br>☐ Hypotension<br>☐ Normotension<br>☐ Other
8.4.36.1.5.2.1	***If 'Response to medication' is equal to 'Other' answer this question:***<br>Other response	
8.4.36.1.6	***If 'How many surgical interventions?' is equal to 'One' answer this question:***<br>Outcome of surgical exicision	○ Radical<br>○ Irradical<br>○ Unknown
8.4.36.1.7	***If 'How many surgical interventions?' is equal to 'One' answer this question:***<br>PA available?	○ Yes<br>○ No
8.4.36.1.7.1	***If 'PA available?' is equal to 'Yes' answer this question:***<br>PA number of specimen	
8.4.36.1.8	***If 'How many surgical interventions?' is equal to 'One' answer this question:***<br>Postoperative complications	☐ Yes<br>☐ No<br>☐ Unknown
8.4.36.1.8.1	***If 'Postoperative complications' is equal to 'Yes' answer this question:***<br>Specification of postoperative complications	☐ Death<br>☐ Stroke<br>☐ Permanent cranial nerve injury<br>☐ Temporary cranial nerve injury<br>☐ Neurologic deficit<br>☐ Infection<br>☐ Hemorrhage/hematoma<br>☐ Other
8.4.36.1.8.1.1	***If 'Specification of postoperative complications' is equal to 'Permanent cranial nerve injury' answer this question:***<br>Permanent damage of cranial nerves	☐ n. IX<br>☐ n. X<br>☐ n. XI<br>☐ n. XII<br>☐ n. VII<br>☐ n. VIII

8.4.36.1.8.1.2	***If 'Specification of postoperative complications' is equal to 'Temporary cranial nerve injury' answer this question:***<br>Temporary damage of cranial nerves	☐ n. IX<br>☐ n. X<br>☐ n. XI<br>☐ n. XII<br>☐ n. VII<br>☐ n. VIII
8.4.36.1.8.1.3	***If 'Specification of postoperative complications' is equal to 'Neurologic deficit' answer this question:***<br>Neurological deficit	☐ Horner Syndrome<br>☐ First bite syndrome<br>☐ Central nerve deficit<br>☐ Peripheral nerve deficit<br>☐ Laryngeus recurrens<br>☐ Other
8.4.36.1.8.1.4	***If 'Specification of postoperative complications' is equal to 'Other' answer this question:***<br>Other surigcal complications	
	NOTE: please name surgical intervention as e.g.: SI_dd_mm_yyyy	
8.4.36.1.10	***If 'How many surgical interventions?' is equal to 'Multiple' answer this question:***<br>Specification of all PGL surgical interventions	
8.4.37	***If 'All diagnosed paraganglioma' is not equal to 'None' answer this question:***<br>Embolization of a paraganglioma?	○ Yes<br>○ No<br>○ Unknown
8.4.37.1	***If 'Embolization of a paraganglioma?' is equal to 'Yes' answer this question:***<br>Embolization of specific paraganglioma	☐ Carotid body left<br>☐ Carotid body right<br>☐ Tympanic left<br>☐ Tympanic right<br>☐ Jugular left<br>☐ Jugular right<br>☐ Vagal left<br>☐ Vagal right<br>☐ Thoracic<br>☐ Abdominal<br>☐ Pheochromocytoma<br>☐ Other<br>☐ None<br>☐ Unknown
8.4.37.2	***If 'Embolization of a paraganglioma?' is equal to 'Yes' answer this question:***<br>Complications due to embolization?	○ Yes<br>○ No<br>○ Unknown

	NOTE: please name radiotherapy as: RT_paragangliomalocation_dd_mm_yyyy	
8.4.39	***If 'All diagnosed paraganglioma' is not equal to 'None' answer this question:***<br>Radiotherapy or PRRT received for paraganglioma?	○ Yes<br>○ No
8.4.39.1	***If 'Radiotherapy or PRRT received for paraganglioma?' is equal to 'Yes' answer this question:***<br>Specification of all radiotherapy treatments	

9. Follow-up - Yearly check up

Number	Question	Answers
	NOTE: these questions are only for data obtained from monthly/yearly check ups when patients course of disease changes significantly.	
9.1	Change in course of disease?	◯ Yes<br>◯ No
9.1.1	***If 'Change in course of disease?' is equal to 'Yes' answer this question:***<br>Date of follow up appointment<br>*Warning shown if field's value is larger than NOW: 'Date cannot be in the future.'*	[ ] [ ] [ ] *(dd-mm-yyyy)*
9.1.2	***If 'Change in course of disease?' is equal to 'Yes' answer this question:***<br>Symptoms	☐ Swelling in the neck<br>☐ Hoarseness<br>☐ Pain<br>☐ Difficulty swallowing<br>☐ Tinnitus<br>☐ Hearing loss<br>☐ Palpitations<br>☐ Excessive sweating<br>☐ Incidentaloma<br>☐ Dizziness<br>☐ Visual complaints<br>☐ Coughing<br>☐ Malignant hypertension<br>☐ Agitated<br>☐ No complaints<br>☐ Other<br>☐ Unknown
9.1.2.1	***If 'Symptoms' is equal to 'Other' answer this question:***<br>What other symptoms?	[ ]
9.1.3	***If 'Change in course of disease?' is equal to 'Yes' answer this question:***<br>Hormone production	◯ Yes<br>◯ No<br>◯ Unknown
9.1.4	***If 'Change in course of disease?' is equal to 'Yes' answer this question:***<br>Tumor recurrence?	☐ Yes<br>☐ No

9.1.4.1	***If 'Tumor recurrence?' is equal to 'Yes' answer this question:***<br>Recurrence of which paraganglioma?	☐ Carotid body left<br>☐ Carotid body right<br>☐ Tympanic left<br>☐ Tympanic right<br>☐ Jugular left<br>☐ Jugular right<br>☐ Vagal left<br>☐ Vagal right<br>☐ Thoracic<br>☐ Abdominal<br>☐ Pheochromocytoma<br>☐ Other<br>☐ None<br>☐ Unknown
9.1.4.1.1	***If 'Recurrence of which paraganglioma?' is equal to 'Other' answer this question:***<br>What other paraganglioma?	
9.1.5	***If 'Change in course of disease?' is equal to 'Yes' answer this question:***<br>Malignant tumor transformation?	○ Yes<br>○ No
9.1.6	***If 'Change in course of disease?' is equal to 'Yes' answer this question:***<br>Additional paraganglioma(s)	○ Yes<br>○ No
9.1.6.1	***If 'Additional paraganglioma(s)' is equal to 'Yes' answer this question:***<br>Location of additional paraganglioma(s)	☐ Carotid body left<br>☐ Carotid body right<br>☐ Tympanic left<br>☐ Tympanic right<br>☐ Jugular left<br>☐ Jugular right<br>☐ Vagal left<br>☐ Vagal right<br>☐ Thoracic<br>☐ Abdominal<br>☐ Pheochromocytoma<br>☐ Other<br>☐ None<br>☐ Unknown

9.1.6.1.1	***If 'Location of additional paraganglioma(s)' is equal to 'Thoracic' answer this question:*** Specification of thoracic paraganglioma	☐ Pulmonary<br>☐ Mediastinal<br>☐ Diaphragmatic<br>☐ Spine<br>☐ Para aortal<br>☐ Cardiac<br>☐ Aorticopulmonary<br>☐ Para tracheal<br>☐ Other
9.1.6.1.1.1	***If 'Specification of thoracic paraganglioma' is equal to 'Other' answer this question:*** Specification of other thoracic paraganglioma	
9.1.6.1.2	***If 'Location of additional paraganglioma(s)' is equal to 'Abdominal' answer this question:*** Specification of abdominal paraganglioma	☐ Mesenteric<br>☐ Para aortal<br>☐ Organ of Zuckerkandl<br>☐ Duodenal/ ampulla of Vater<br>☐ Bladder<br>☐ Colorectal<br>☐ Retroperitoneal<br>☐ Gonadal<br>☐ Peri-renal<br>☐ Sympathetic trunc<br>☐ Other
9.1.6.1.2.1	***If 'Specification of abdominal paraganglioma' is equal to 'Other' answer this question:*** Specification of other abdominal paraganglioma	
9.1.6.1.3	***If 'Location of additional paraganglioma(s)' is equal to 'Pheochromocytoma' answer this question:*** Specification of pheochromocytoma	☐ Left adrenal gland<br>☐ Right adrenal gland<br>☐ Other
9.1.6.1.3.1	***If 'Specification of pheochromocytoma' is equal to 'Other' answer this question:*** Specification of other pheochromocytoma	
9.1.6.1.4	***If 'Location of additional paraganglioma(s)' is equal to 'Other' answer this question:*** Location of other paraganglioma	
	NOTE: If patient experienced several episodes of changes in course of disease, please fill in all episodes in the field below as "Follow-Up yyyy-mm-dd"	
9.1.8	***If 'Change in course of disease?' is equal to 'Yes' answer this question:*** Add additional episodes of change in course of disease	

10. Follow-up - Urine results

Number	Question	Answers
10.1	***If 'Urine sample obtained?' is equal to 'Yes' answer this question:***<br>Additional urine sample(s) obtainened?	○ Yes<br>○ No
	NOTE: follow up data for additional POSITIVE urine samples, obtained after the first hospital visit.	
10.1.2	***If 'Additional urine sample(s) obtainened?' is equal to 'Yes' answer this question:***<br>Urine sample positive for metanephrines/catecholamines?	○ Yes<br>○ No
	NOTE: please name additional urine samples s as: US_date -> via 'Add Measurements' button	
10.1.2.2	***If 'Urine sample positive for metanephrines/catecholamines?' is equal to 'Yes' answer this question:***<br>Urine sample for catecholamines/metanephrines	

11. Follow-up - Blood results

Number	Question	Answers
11.1	***If 'Blood test obtained?' is equal to 'Yes' answer this question:***<br>Additional blood sample(s) obtained?	◯ Yes<br>◯ No
	NOTE: follow up data for additional POSITIVE blood samples, obtained after the first hospital visit.	
11.1.2	***If 'Additional blood sample(s) obtained?' is equal to 'Yes' answer this question:***<br>Blood results positive for metanephrines/catecholamines?	◯ Yes<br>◯ No
	NOTE: please name additional blood samples: BS_date -> via 'Add Measurement' button	
11.1.2.2	***If 'Blood results positive for metanephrines/catecholamines?' is equal to 'Yes' answer this question:***<br>Blood results catecholamines/metanephrines	

12. Follow-up - Imaging techniques

Number	Question	Answers
	NOTE: add here all new imaging diagnostics belonging to the follow up	
12.1	Additional diagnostics obtained?	◯ Yes<br>◯ No
	NOTE: please name additional scans as: TypeScan_specificarea_date -> via 'Add Report' button e.g. MRI_wholebody_yyyymmdd	
12.1.2	***If 'Additional diagnostics obtained?' is equal to 'Yes' answer this question:***<br>Add additional scans	

13. Outcome - Outcome measures

Number	Question	Answers
13.1	Status	◯ Under follow-up<br>◯ Referred back to GP<br>◯ Lost to follow-up<br>◯ Death<br>◯ Referred to other center
13.1.1	***If 'Status' is equal to 'Referred to other center' answer this question:***<br>Referral to what type of center?	◯ Primary level<br>◯ Secondary level<br>◯ Tertiary level<br>◯ Nursing home<br>◯ Rehabilitation clinic<br>◯ Other
13.1.1.1	***If 'Referral to what type of center?' is equal to 'Other' answer this question:***<br>What other center	
13.1.2	***If 'Status' is equal to 'Lost to follow-up' answer this question:***<br>Date lost to follow-up<br>*Warning shown if field's value is larger than NOW: 'Date is in the future!'*	*(dd-mm-yyyy)*
13.1.3	***If 'Status' is equal to 'Lost to follow-up' answer this question:***<br>Reason for lost to follow-up	☐ Unreachable<br>☐ Error in computer tracking system<br>☐ Financial problems<br>☐ Transportation problems<br>☐ Work responsibilities<br>☐ Child responsibilities<br>☐ Too much work filling in questionaires<br>☐ Bad treatment outcome<br>☐ No reason known<br>☐ Other
13.1.3.1	***If 'Reason for lost to follow-up' is equal to 'Other' answer this question:***<br>Other reason lost to follow up	

14. Research - Follow Up

Number	Question	Answers
14.1	Patient status	◯ Alive with disease (AWD)<br>◯ No evidence of disease (NED)<br>◯ Death intercurrent disease (DID)<br>◯ Death of disease (DOD)<br>◯ Lost to follow up (LTF)
14.1.1	***If 'Patient status' is equal to 'Death intercurrent disease (DID)' answer this question:***<br>Date of death	(dd-mm-yyyy)
14.1.2	***If 'Patient status' is equal to 'Death of disease (DOD)' answer this question:***<br>Date of death	(dd-mm-yyyy)
14.1.3	***If 'Patient status' is equal to 'Death intercurrent disease (DID)' answer this question:***<br>Death due to what other cause?	
14.1.4	***If 'Patient status' is equal to 'Lost to follow up (LTF)' answer this question:***<br>Reason lost to follow up (LTF)	
14.2	Date last out patient clinic<br>*Exclude patient if field's value is larger than NOW with message: 'Date cannot be in the future.'*	(dd-mm-yyyy)
14.3	Other comments	

Reports of Head and Neck Paraganglioma Registry - version 233.91

Printed on 06-12-2023 19:30:32 by Carolijn de Bresser

Repeating Data 'Lab results'

Form Catecholamines/Metanephrines

Number	Question	Answers
1.1	Date of blood sample *Exclude patient if field's value is larger than NOW with message: 'Date of blood sample cannot be in the future.'*	(dd-mm-yyyy)
1.2	Measurement position	◯ Lying down<br>◯ Sitting<br>◯ Standing<br>◯ Unknown
1.3	Does the patient use any interfering medication during blood withdrawel?	◯ Yes<br>◯ No<br>◯ Unknown
1.3.1	***If 'Does the patient use any interfering medication during blood withdrawel?' is equal to 'Yes' answer this question:***<br>What medication does the patient use?	☐ SSRI's (Selective Serotonine Reuptake Inhibitor)<br>☐ TCA (Tricyclic antidepressant)<br>☐ MAO inhibitor (Monoamine Oxidase)<br>☐ Ethanol<br>☐ Other
1.3.1.1	***If 'What medication does the patient use?' is equal to 'Other' answer this question:***<br>Other medication	
1.4	Free 3-MT concentration	nmol/L
	Upper limit for normal range is reached	
1.5	Free Metanephrine concentration	nmol/L
	Upper limit for normal range is reached	
1.6	Free Normetanephrine (NM) concentration	nmol/L
	Upper limit for normal range is reached	
1.7	Postive blood serum upon absolute values	
1.7.1	***If 'Postive blood serum upon absolute values' is equal to 'Yes' answer this question:***<br>Blood sample drawn under ideal conditions?	◯ Yes<br>◯ No<br>◯ Unknown

1.7.2	***If 'Postive blood serum upon absolute values' is equal to 'Yes' answer this question:***<br>Experiencing symptoms	☐ Headache<br>☐ Excessive sweating<br>☐ Paleness<br>☐ Anxiety<br>☐ Tremor<br>☐ Shortness of breath<br>☐ High blood pressure<br>☐ Rapid heart beat<br>☐ Exhaustion/tiredness<br>☐ Rushed feeling<br>☐ Other<br>☐ None<br>☐ Unknown
1.7.2.1	***If 'Experiencing symptoms' is equal to 'High blood pressure' answer this question:***<br>Systolic blood pressure	[ ] mmHg
1.7.2.2	***If 'Experiencing symptoms' is equal to 'High blood pressure' answer this question:***<br>Diastolic blood pressure	[ ] mmHg
1.7.2.3	***If 'Experiencing symptoms' is equal to 'Rapid heart beat' answer this question:***<br>Heart rate	[ ] beats/min
1.7.2.4	***If 'Experiencing symptoms' is equal to 'Other' answer this question:***<br>What other symptoms?	[ ]
1.7.3	***If 'Postive blood serum upon absolute values' is equal to 'Yes' answer this question:***<br>Medicinal treatment obtained for symptoms?	○ Yes<br>○ No<br>○ Unknown
1.7.3.1	***If 'Medicinal treatment obtained for symptoms?' is equal to 'Yes' answer this question:***<br>What type of medicinal treatment?	○ Alpha blocker<br>○ Beta blocker<br>○ Calcium channel antagonist<br>○ Diuretics<br>○ Vasodilators<br>○ ACE inhitibors<br>○ Benzodiazepines<br>○ Other
1.7.3.1.1	***If 'What type of medicinal treatment?' is equal to 'Other' answer this question:***<br>Other medicinal treatment	[ ]
1.7.3.2	***If 'Medicinal treatment obtained for symptoms?' is equal to 'Yes' answer this question:***	○ Therapy resistant

	Response to medication	◯ Hypotension<br>◯ Normotension<br>◯ Other
1.7.3.2.1	***If 'Response to medication' is equal to 'Other' answer this question:***<br>Other response	
1.7.3.3	***If 'Medicinal treatment obtained for symptoms?' is equal to 'Yes' answer this question:***<br>Normalisation of hormones through treatment?	◯ Yes<br>◯ No<br>◯ Unknown<br>◯ No treatment obtained
1.7.1.1	***If 'Blood sample drawn under ideal conditions?' is equal to 'No' answer this question:***<br>Repetition of blood withdrawel under ideal conditions	◯ Yes<br>◯ No<br>◯ Unknown
1.7.1.1.1	***If 'Repetition of blood withdrawel under ideal conditions' is equal to 'Yes' answer this question:***<br>Normalisation of hormones after blood drawn under ideal conditions?	◯ Yes<br>◯ No<br>◯ Unknown

Repeating Data 'Blood pressure'

Form BP measurements

Number	Question	Answers
1.1	Date and time of measurement	☐ ☐ ☐ *(dd-mm-yyyy)*<br>☐ : ☐ *(hh:mm)*
1.2	Measurement position	◯ Lying down<br>◯ Sitting<br>◯ Standing<br>◯ Unknown
1.3	Systolic pressure	☐ mmHg
1.4	Diastolic pressure	☐ mmHg

Repeating Data 'Malignant paraganglioma'

Form Malignant paraganglioma

Number	Question	Answers
1.1	Date of diagnosis	☐ ☐ ☐ *(dd-mm-yyyy)*
1.2	Location of malignant paraganglioma	☐ Carotid body left<br>☐ Carotid body right<br>☐ Tympanic left<br>☐ Tympanic right<br>☐ Jugular left<br>☐ Jugular right<br>☐ Vagal left<br>☐ Vagal right<br>☐ Thoracic<br>☐ Abdominal<br>☐ Pheochromocytoma<br>☐ Other<br>☐ None<br>☐ Unknown
1.2.1	***If 'Location of malignant paraganglioma' is equal to 'Thoracic' answer this question:***<br>Thoracic location	○ Pulmonary<br>○ Mediastinal<br>○ Diaphragmatic<br>○ Spine<br>○ Para aortal<br>○ Cardiac<br>○ Aorticopulmonary<br>○ Para tracheal<br>○ Other
1.2.2	***If 'Location of malignant paraganglioma' is equal to 'Abdominal' answer this question:***<br>Abdominal location	○ Mesenteric<br>○ Para aortal<br>○ Organ of Zuckerkandl<br>○ Duodenal/ ampulla of Vater<br>○ Bladder<br>○ Colorectal<br>○ Retroperitoneal<br>○ Gonadal<br>○ Peri-renal<br>○ Sympathetic trunc<br>○ Other

1.2.3	***If 'Location of malignant paraganglioma' is equal to 'Pheochromocytoma' answer this question:***<br>Pheochromocytoma location	◯ Left adrenal gland<br>◯ Right adrenal gland<br>◯ Other
1.3	Location of metastasis/metastases	☐ Locoregional<br>☐ Distant
1.3.1	***If 'Location of metastasis/metastases' is equal to 'Locoregional' answer this question:***<br>Locoregional metastases location(s)	☐ Vessel(s)<br>☐ Nerve(s)<br>☐ Lymph node(s)<br>☐ Surrounding tissue<br>☐ Other
1.3.1.1	***If 'Locoregional metastases location(s)' is equal to 'Other' answer this question:***<br>Other location	[ ]
1.3.2	***If 'Location of metastasis/metastases' is equal to 'Distant' answer this question:***<br>Distant metastases location(s)	☐ Bone<br>☐ Liver<br>☐ Lung<br>☐ Brain<br>☐ Adrenal gland<br>☐ Peritoneum<br>☐ Lymph node<br>☐ Skin<br>☐ Other
1.3.2.1	***If 'Distant metastases location(s)' is equal to 'Other' answer this question:***<br>Other location	[ ]
1.4	Treatment of malignant pheochromocytoma/paraganglioma	◯ Surgery<br>◯ Wait-and-scan<br>◯ Radiotherapy<br>◯ Medication<br>◯ Other<br>◯ None
1.4.1	***If 'Treatment of malignant pheochromocytoma/paraganglioma' is not equal to 'None' answer this question:***<br>Date of treatment	[ ] [ ] [ ] *(dd-mm-yyyy)*
1.5	PA available?	◯ Yes<br>◯ No
1.5.1	***If 'PA available?' is equal to 'Yes' answer this question:***<br>PA number	[ ] 20

1.6	Complications due to treatment	◯ Yes<br>◯ No
1.6.1	***If 'Complications due to treatment' is equal to 'Yes' answer this question:***<br>Specification of complications	◯ Death<br>◯ Stroke<br>◯ Permanent cranial nerve injury<br>◯ Temporary cranial nerve injury<br>◯ Neurologic deficit<br>◯ Infection<br>◯ Hemorrhage/hematoma<br>◯ Other
1.6.1.1	***If 'Specification of complications' is equal to 'Other' answer this question:***<br>Other complications	

Repeating Data 'Urine sample'

Form Urine sample

Number	Question	Answers
	All urine samples from before current visit, and future follow-up	
1.1	Date of urine sample *Exclude patient if field's value is larger than NOW with message: 'Date cannot be in the future.'*	☐ ☐ ☐ *(dd-mm-yyyy)*
1.2	Vanillylmandelic acid / 24 h	☐ umol/24hours
1.3	Metanephrines / 24 h	☐ umol/24h
1.4	Normetanephrine / 24 h	☐ umol/24h
1.5	Free adrenaline (epinephrine) / 24h	☐ nmol/24 h
1.6	Free noradrenaline (norepinephrine) / 24h	☐ nmol/24 h
1.7	Free dopamine / 24h	☐ nmol/24h
1.8	Positive urine sample?	
1.8.1	***If 'Positive urine sample?' is equal to 'Yes' answer this question:***<br>Symptoms/complaints due to positive urine sample?	○ Yes<br>○ No<br>○ Unknown
1.8.1.1	***If 'Symptoms/complaints due to positive urine sample?' is equal to 'Yes' answer this question:***<br>Experienced symptoms	☐ Headache<br>☐ Excessive sweating<br>☐ Paleness<br>☐ Anxiety<br>☐ Tremor<br>☐ Shortness of breath<br>☐ High blood pressure<br>☐ Rapid heart beat<br>☐ Exhaustion/tiredness<br>☐ Rushed feeling<br>☐ Other<br>☐ None<br>☐ Unknown
1.8.1.1.1	***If 'Experienced symptoms' is equal to 'Other' answer this question:***<br>Other symptoms	☐
1.8.2	***If 'Positive urine sample?' is equal to 'Yes' answer this question:***	○ Yes

	Medicinal treatment after elevated urine hormones?	◯ No<br>◯ Unknown
1.8.2.1	***If 'Medicinal treatment after elevated urine hormones?' is equal to 'Yes' answer this question:***<br>What type of medicinal treatment?	☐ Alpha blocker<br>☐ Beta blocker<br>☐ Calcium channel antagonist<br>☐ Diuretics<br>☐ Vasodilators<br>☐ ACE inhitibors<br>☐ Benzodiazepines<br>☐ Other
1.8.2.2	***If 'Medicinal treatment after elevated urine hormones?' is equal to 'Yes' answer this question:***<br>Response to medication	◯ Therapy resistant<br>◯ Hypotension<br>◯ Normotension<br>◯ Other
1.8.2.2.1	***If 'Response to medication' is equal to 'Other' answer this question:***<br>Other response	
1.8.3	***If 'Positive urine sample?' is equal to 'Yes' answer this question:***<br>Normalisation of hormones in urine sample?	◯ Yes<br>◯ No<br>◯ Unknown<br>◯ No treatment obtained

Repeating Data 'Imaging Techniques'

Form Imaging Techniques

Number	Question	Answers
	NOTE: only fill in the results of the scan when they are different from the previous scan	
1.1	Imaging techniques	☐ Ultrasound (US) of the neck<br>☐ US of the neck with biopsy<br>☐ CT of the head/neck<br>☐ CT-angiography<br>☐ CT-thorax<br>☐ CT-abdomen<br>☐ MRI of the head/neck<br>☐ MRI paraganglioma (whole body)<br>☐ FDG-PET<br>☐ MIBG<br>☐ F-DOPA<br>☐ 68Ga-DOTA scan<br>☐ Other<br>☐ None<br>☐ Other hospital
1.1.1	***If 'Imaging techniques' is equal to 'Ultrasound (US) of the neck' answer this question:***<br>Date of head/neck US *Warning shown if field's value is larger than NOW: 'Date cannot be in the future'*	___ ___ ___ *(dd-mm-yyyy)*
1.1.2	***If 'Imaging techniques' is equal to 'Ultrasound (US) of the neck' answer this question:***<br>Abnormalities on ultrasound?	○ Yes<br>○ No

1.1.2.1	***If 'Abnormalities on ultrasound?' is equal to 'Yes' answer this question:***<br>Detected paragangliomas	☐ Carotid body left<br>☐ Carotid body right<br>☐ Tympanic left<br>☐ Tympanic right<br>☐ Jugular left<br>☐ Jugular right<br>☐ Vagal left<br>☐ Vagal right<br>☐ Thoracic<br>☐ Abdominal<br>☐ Pheochromocytoma<br>☐ Other<br>☐ None<br>☐ Unknown
1.1.2.1.1	***If 'Detected paragangliomas' is equal to 'Thoracic' answer this question:***<br>Specification of thoracic paraganglioma	☐ Pulmonary<br>☐ Mediastinal<br>☐ Diaphragmatic<br>☐ Spine<br>☐ Para aortal<br>☐ Cardiac<br>☐ Aorticopulmonary<br>☐ Para tracheal<br>☐ Other
1.1.2.1.2	***If 'Detected paragangliomas' is equal to 'Abdominal' answer this question:***<br>Specification of abdominal paraganglioma	☐ Mesenteric<br>☐ Para aortal<br>☐ Organ of Zuckerkandl<br>☐ Duodenal/ ampulla of Vater<br>☐ Bladder<br>☐ Colorectal<br>☐ Retroperitoneal<br>☐ Gonadal<br>☐ Peri-renal<br>☐ Sympathetic trunc<br>☐ Other
1.1.2.1.3	***If 'Detected paragangliomas' is equal to 'Pheochromocytoma' answer this question:***<br>Location of pheochromocytoma	☐ Left adrenal gland<br>☐ Right adrenal gland<br>☐ Other
1.1.2.1.4	***If 'Detected paragangliomas' is equal to 'Other' answer this question:***<br>Location of other paraganglioma	______________
1.1.2.2	***If 'Abnormalities on ultrasound?' is equal to 'Yes' answer this question:***	☐ Growth

	Change compared to the previous US	☐ Additional tumor<br>☐ Metastasis/metastases<br>☐ Ingrow in surrounding structure(s)<br>☐ Other<br>☐ None
1.1.2.2.1	***If 'Change compared to the previous US' is equal to 'Other' answer this question:***<br>Other changes	[ ]
1.1.3	***If 'Imaging techniques' is equal to 'US of the neck with biopsy' answer this question:***<br>Date of head/neck US + biopsy *Warning shown if field's value is larger than NOW: 'Date cannot be in the future'*	[ ] [ ] [ ] *(dd-mm-yyyy)*
1.1.4	***If 'Imaging techniques' is equal to 'US of the neck with biopsy' answer this question:***<br>Outcome of biopsy	○ Paraganglioma<br>○ Normal tissue from biopsy place<br>○ Other malignancy
1.1.5	***If 'Imaging techniques' is equal to 'US of the neck with biopsy' answer this question:***<br>Abnormalities on ultrasound?	○ Yes<br>○ No
1.1.5.1	***If 'Abnormalities on ultrasound?' is equal to 'Yes' answer this question:***<br>Detected paragangliomas on US	☐ Carotid body left<br>☐ Carotid body right<br>☐ Tympanic left<br>☐ Tympanic right<br>☐ Jugular left<br>☐ Jugular right<br>☐ Vagal left<br>☐ Vagal right<br>☐ Thoracic<br>☐ Abdominal<br>☐ Pheochromocytoma<br>☐ Other<br>☐ None<br>☐ Unknown
1.1.5.1.1	***If 'Detected paragangliomas on US' is equal to 'Thoracic' answer this question:***<br>Specification of thoracic paraganglioma	☐ Pulmonary<br>☐ Mediastinal<br>☐ Diaphragmatic<br>☐ Spine<br>☐ Para aortal<br>☐ Cardiac<br>☐ Aorticopulmonary<br>☐ Para tracheal<br>☐ Other
1.1.5.1.2	***If 'Detected paragangliomas on US' is equal to***	☐ Mesenteric

	'Abdominal' answer this question: Specification of abdominal paraganglioma	☐ Para aortal<br>☐ Organ of Zuckerkandl<br>☐ Duodenal/ ampulla of Vater<br>☐ Bladder<br>☐ Colorectal<br>☐ Retroperitoneal<br>☐ Gonadal<br>☐ Peri-renal<br>☐ Sympathetic trunc<br>☐ Other
1.1.5.1.3	***If 'Detected paragangliomas on US' is equal to 'Pheochromocytoma' answer this question:*** Location of pheochromocytoma	☐ Left adrenal gland<br>☐ Right adrenal gland<br>☐ Other
1.1.5.1.4	***If 'Detected paragangliomas on US' is equal to 'Other' answer this question:*** Location of other paraganglioma	
1.1.5.2	***If 'Abnormalities on ultrasound?' is equal to 'Yes' answer this question:*** Change compared to the previous US	☐ Growth<br>☐ Additional tumor<br>☐ Metastasis/metastases<br>☐ Ingrow in surrounding structure(s)<br>☐ Other<br>☐ None
1.1.5.2.1	***If 'Change compared to the previous US' is equal to 'Other' answer this question:*** Other changes	
1.1.6	***If 'Imaging techniques' is equal to 'CT of the head/neck' answer this question:*** Date of head/neck CT	*(dd-mm-yyyy)*
1.1.7	***If 'Imaging techniques' is equal to 'CT of the head/neck' answer this question:*** Abnormalities on head/neck CT?	○ Yes<br>○ No
1.1.7.1	***If 'Abnormalities on head/neck CT?' is equal to 'Yes' answer this question:*** Detected paragangliomas	☐ Carotid body left<br>☐ Carotid body right<br>☐ Tympanic left<br>☐ Typmanic right<br>☐ Jugular left<br>☐ Jugular right<br>☐ Vagal left<br>☐ Vagal right<br>☐ Other<br>☐ Unknown

1.1.7.2	***If 'Abnormalities on head/neck CT?' is equal to 'Yes' answer this question:***<br>Change compared to the previous head/neck CT	☐ Growth<br>☐ Additional tumor<br>☐ Metastasis/metastases<br>☐ Ingrow in surrounding structure(s)<br>☐ Other<br>☐ None
1.1.7.2.1	***If 'Change compared to the previous head/neck CT' is equal to 'Other' answer this question:***<br>Other changes	
1.1.8	***If 'Imaging techniques' is equal to 'CT-angiography' answer this question:***<br>Date of CT angiography	*(dd-mm-yyyy)*
1.1.9	***If 'Imaging techniques' is equal to 'CT-angiography' answer this question:***<br>Abnormalities on angiography?	○ Yes<br>○ No
1.1.9.1	***If 'Abnormalities on angiography?' is equal to 'Yes' answer this question:***<br>Detected paragangliomas	☐ Carotid body left<br>☐ Carotid body right<br>☐ Tympanic left<br>☐ Tympanic right<br>☐ Jugular left<br>☐ Jugular right<br>☐ Vagal left<br>☐ Vagal right<br>☐ Thoracic<br>☐ Abdominal<br>☐ Pheochromocytoma<br>☐ Other<br>☐ None<br>☐ Unknown
1.1.9.1.1	***If 'Detected paragangliomas' is equal to 'Thoracic' answer this question:***<br>Specification of thoracic paraganglioma	☐ Pulmonary<br>☐ Mediastinal<br>☐ Diaphragmatic<br>☐ Spine<br>☐ Para aortal<br>☐ Cardiac<br>☐ Aorticopulmonary<br>☐ Para tracheal<br>☐ Other
1.1.9.1.2	***If 'Detected paragangliomas' is equal to 'Abdominal' answer this question:***<br>Specification of abdominal paraganglioma	☐ Mesenteric<br>☐ Para aortal<br>☐ Organ of Zuckerkandl<br>☐ Duodenal/ ampulla of Vater

		☐ Bladder<br>☐ Colorectal<br>☐ Retroperitoneal<br>☐ Gonadal<br>☐ Peri-renal<br>☐ Sympathetic trunc<br>☐ Other
1.1.9.1.3	***If 'Detected paragangliomas' is equal to 'Pheochromocytoma' answer this question:***<br>Location of pheochromocytoma	☐ Left adrenal gland<br>☐ Right adrenal gland<br>☐ Other
1.1.9.1.4	***If 'Detected paragangliomas' is equal to 'Other' answer this question:***<br>Location of other paraganglioma	
1.1.9.2	***If 'Abnormalities on angiography?' is equal to 'Yes' answer this question:***<br>Change compared to the previous CT angiography	☐ Growth<br>☐ Additional tumor<br>☐ Metastasis/metastases<br>☐ Ingrow in surrounding structure(s)<br>☐ Other<br>☐ None
1.1.9.2.1	***If 'Change compared to the previous CT angiography' is equal to 'Other' answer this question:***<br>Other changes	
1.1.10	***If 'Imaging techniques' is equal to 'CT-thorax' answer this question:***<br>Date of thoracic CT	*(dd-mm-yyyy)*
1.1.11	***If 'Imaging techniques' is equal to 'CT-thorax' answer this question:***<br>Abnormalities on thoracic CT?	○ Yes<br>○ No
1.1.11.1	***If 'Abnormalities on thoracic CT?' is equal to 'Yes' answer this question:***<br>Specification of thoracic paraganglioma	☐ Pulmonary<br>☐ Mediastinal<br>☐ Diaphragmatic<br>☐ Spine<br>☐ Para aortal<br>☐ Cardiac<br>☐ Aorticopulmonary<br>☐ Para tracheal<br>☐ Other
1.1.11.2	***If 'Abnormalities on thoracic CT?' is equal to 'Yes' answer this question:***<br>Change compared to the previous thoracic CT	☐ Growth<br>☐ Additional tumor<br>☐ Metastasis/metastases<br>☐ Ingrow in surrounding structure(s)

		☐ Other<br>☐ None
1.1.11.2.1	***If 'Change compared to the previous thoracic CT' is equal to 'Other' answer this question:***<br>Other changes	
1.1.12	***If 'Imaging techniques' is equal to 'CT-abdomen' answer this question:***<br>Date of abdominal CT?	_ _ _ *(dd-mm-yyyy)*
1.1.13	***If 'Imaging techniques' is equal to 'CT-abdomen' answer this question:***<br>Abnormalities on abdominal CT?	○ Yes<br>○ No
1.1.13.1	***If 'Abnormalities on abdominal CT?' is equal to 'Yes' answer this question:***<br>Specification of abdominal paraganglioma	☐ Mesenteric<br>☐ Para aortal<br>☐ Organ of Zuckerkandl<br>☐ Duodenal/ ampulla of Vater<br>☐ Bladder<br>☐ Colorectal<br>☐ Retroperitoneal<br>☐ Gonadal<br>☐ Peri-renal<br>☐ Sympathetic trunc<br>☐ Other
1.1.13.2	***If 'Abnormalities on abdominal CT?' is equal to 'Yes' answer this question:***<br>Change compared to the previous abdominal CT	☐ Growth<br>☐ Additional tumor<br>☐ Metastasis/metastases<br>☐ Ingrow in surrounding structure(s)<br>☐ Other<br>☐ None
1.1.13.2.1	***If 'Change compared to the previous abdominal CT' is equal to 'Other' answer this question:***<br>Other changes	
1.1.14	***If 'Imaging techniques' is equal to 'MRI of the head/neck' answer this question:***<br>Date of head/neck MRI	_ _ _ *(dd-mm-yyyy)*
1.1.15	***If 'Imaging techniques' is equal to 'MRI of the head/neck' answer this question:***<br>Abnormalities on head/neck MRI?	○ Yes<br>○ No
1.1.15.1	***If 'Abnormalities on head/neck MRI?' is equal to 'Yes' answer this question:***<br>Detected paragangliomas on head/neck MRI	☐ Carotid body left<br>☐ Carotid body right<br>☐ Tympanic left<br>☐ Typmanic right<br>☐ Jugular left

		☐ Jugular right<br>☐ Vagal left<br>☐ Vagal right<br>☐ Other<br>☐ Unknown
1.1.15.2	***If 'Abnormalities on head/neck MRI?' is equal to 'Yes' answer this question:***<br>Change compared to the previous head/neck MRI	☐ Growth<br>☐ Additional tumor<br>☐ Metastasis/metastases<br>☐ Ingrow in surrounding structure(s)<br>☐ Other<br>☐ None
1.1.15.2.1	***If 'Change compared to the previous head/neck MRI' is equal to 'Other' answer this question:***<br>Other changes	[ ]
1.1.16	***If 'Imaging techniques' is equal to 'MRI paraganglioma (whole body)' answer this question:***<br>Date of MRI paraganglioma (whole body)	[ ] [ ] [ ] *(dd-mm-yyyy)*
1.1.17	***If 'Imaging techniques' is equal to 'MRI paraganglioma (whole body)' answer this question:***<br>Abnormalities on MRI paraganglioma (whole body)?	○ Yes<br>○ No
1.1.17.1	***If 'Abnormalities on MRI paraganglioma (whole body)?' is equal to 'Yes' answer this question:***<br>Detected paragangliomas on MRI whole body	☐ Carotid body left<br>☐ Carotid body right<br>☐ Tympanic left<br>☐ Tympanic right<br>☐ Jugular left<br>☐ Jugular right<br>☐ Vagal left<br>☐ Vagal right<br>☐ Thoracic<br>☐ Abdominal<br>☐ Pheochromocytoma<br>☐ Other<br>☐ None<br>☐ Unknown

1.1.17.1.1	***If 'Detected paragangliomas on MRI whole body' is equal to 'Thoracic' answer this question:***<br>Specification of thoracic paraganglioma	☐ Pulmonary<br>☐ Mediastinal<br>☐ Diaphragmatic<br>☐ Spine<br>☐ Para aortal<br>☐ Cardiac<br>☐ Aorticopulmonary<br>☐ Para tracheal<br>☐ Other
1.1.17.1.2	***If 'Detected paragangliomas on MRI whole body' is equal to 'Abdominal' answer this question:***<br>Specification of abdominal paraganglioma	☐ Mesenteric<br>☐ Para aortal<br>☐ Organ of Zuckerkandl<br>☐ Duodenal/ ampulla of Vater<br>☐ Bladder<br>☐ Colorectal<br>☐ Retroperitoneal<br>☐ Gonadal<br>☐ Peri-renal<br>☐ Sympathetic trunc<br>☐ Other
1.1.17.1.3	***If 'Detected paragangliomas on MRI whole body' is equal to 'Pheochromocytoma' answer this question:***<br>Location of pheochromocytoma	☐ Left adrenal gland<br>☐ Right adrenal gland<br>☐ Other
1.1.17.1.4	***If 'Detected paragangliomas on MRI whole body' is equal to 'Other' answer this question:***<br>Location of other paraganglioma	
1.1.17.2	***If 'Abnormalities on MRI paraganglioma (whole body)?' is equal to 'Yes' answer this question:***<br>Change compared to the previous MRI whole body	☐ Growth<br>☐ Additional tumor<br>☐ Metastasis/metastases<br>☐ Ingrow in surrounding structure(s)<br>☐ Other<br>☐ None
1.1.17.2.1	***If 'Change compared to the previous MRI whole body' is equal to 'Other' answer this question:***<br>Other changes	
1.1.18	***If 'Imaging techniques' is equal to 'FDG-PET' answer this question:***<br>Date of FDG-PET	*(dd-mm-yyyy)*
1.1.19	***If 'Imaging techniques' is equal to 'FDG-PET' answer this question:***<br>Abnormalities on FDG-PET?	◯ Yes<br>◯ No

1.1.19.1	***If 'Abnormalities on FDG-PET?' is equal to 'Yes' answer this question:***<br>Detected paragangliomas on FDG-PET	☐ Carotid body left<br>☐ Carotid body right<br>☐ Tympanic left<br>☐ Tympanic right<br>☐ Jugular left<br>☐ Jugular right<br>☐ Vagal left<br>☐ Vagal right<br>☐ Thoracic<br>☐ Abdominal<br>☐ Pheochromocytoma<br>☐ Other<br>☐ None<br>☐ Unknown
1.1.19.1.1	***If 'Detected paragangliomas on FDG-PET' is equal to 'Thoracic' answer this question:***<br>Specification of thoracic paraganglioma	☐ Pulmonary<br>☐ Mediastinal<br>☐ Diaphragmatic<br>☐ Spine<br>☐ Para aortal<br>☐ Cardiac<br>☐ Aorticopulmonary<br>☐ Para tracheal<br>☐ Other
1.1.19.1.2	***If 'Detected paragangliomas on FDG-PET' is equal to 'Abdominal' answer this question:***<br>Specification of abdominal paraganglioma	☐ Mesenteric<br>☐ Para aortal<br>☐ Organ of Zuckerkandl<br>☐ Duodenal/ ampulla of Vater<br>☐ Bladder<br>☐ Colorectal<br>☐ Retroperitoneal<br>☐ Gonadal<br>☐ Peri-renal<br>☐ Sympathetic trunc<br>☐ Other
1.1.19.1.3	***If 'Detected paragangliomas on FDG-PET' is equal to 'Pheochromocytoma' answer this question:***<br>Location of pheochromocytoma	☐ Left adrenal gland<br>☐ Right adrenal gland<br>☐ Other
1.1.19.1.4	***If 'Detected paragangliomas on FDG-PET' is equal to 'Other' answer this question:***<br>Location of other paraganglioma	
1.1.19.2	***If 'Abnormalities on FDG-PET?' is equal to 'Yes' answer this question:***	☐ Growth

	Change compared to the previous FDG-PET	☐ Additional tumor<br>☐ Metastasis/metastases<br>☐ Ingrow in surrounding structure(s)<br>☐ Other<br>☐ None
1.1.19.2.1	***If 'Change compared to the previous FDG-PET' is equal to 'Other' answer this question:***<br>Other changes	[ ]
1.1.20	***If 'Imaging techniques' is equal to 'MIBG' answer this question:***<br>Date of MIBG	[ ] [ ] [ ] *(dd-mm-yyyy)*
1.1.21	***If 'Imaging techniques' is equal to 'MIBG' answer this question:***<br>Abnormalities on MIBG?	○ Yes<br>○ No
1.1.21.1	***If 'Abnormalities on MIBG?' is equal to 'Yes' answer this question:***<br>Detected paragangliomas on MIBG	☐ Carotid body left<br>☐ Carotid body right<br>☐ Tympanic left<br>☐ Tympanic right<br>☐ Jugular left<br>☐ Jugular right<br>☐ Vagal left<br>☐ Vagal right<br>☐ Thoracic<br>☐ Abdominal<br>☐ Pheochromocytoma<br>☐ Other<br>☐ None<br>☐ Unknown
1.1.21.1.1	***If 'Detected paragangliomas on MIBG' is equal to 'Thoracic' answer this question:***<br>Specification of thoracic paraganglioma	☐ Pulmonary<br>☐ Mediastinal<br>☐ Diaphragmatic<br>☐ Spine<br>☐ Para aortal<br>☐ Cardiac<br>☐ Aorticopulmonary<br>☐ Para tracheal<br>☐ Other
1.1.21.1.2	***If 'Detected paragangliomas on MIBG' is equal to 'Abdominal' answer this question:***<br>Specification of abdominal paraganglioma	☐ Mesenteric<br>☐ Para aortal<br>☐ Organ of Zuckerkandl<br>☐ Duodenal/ ampulla of Vater<br>☐ Bladder<br>☐ Colorectal

		☐ Retroperitoneal<br>☐ Gonadal<br>☐ Peri-renal<br>☐ Sympathetic trunc<br>☐ Other
1.1.21.1.3	***If 'Detected paragangliomas on MIBG' is equal to 'Pheochromocytoma' answer this question:***<br>Location of pheochromocytoma	☐ Left adrenal gland<br>☐ Right adrenal gland<br>☐ Other
1.1.21.1.4	***If 'Detected paragangliomas on MIBG' is equal to 'Other' answer this question:***<br>Location of other paraganglioma	
1.1.21.2	***If 'Abnormalities on MIBG?' is equal to 'Yes' answer this question:***<br>Change compared to the previous MIBG	☐ Growth<br>☐ Additional tumor<br>☐ Metastasis/metastases<br>☐ Ingrow in surrounding structure(s)<br>☐ Other<br>☐ None
1.1.21.2.1	***If 'Change compared to the previous MIBG' is equal to 'Other' answer this question:***<br>Other changes	
1.1.22	***If 'Imaging techniques' is equal to 'F-DOPA' answer this question:***<br>Date of F-DOPA	*(dd-mm-yyyy)*
1.1.23	***If 'Imaging techniques' is equal to 'F-DOPA' answer this question:***<br>Abnormalities on F-DOPA	○ Yes<br>○ No
1.1.23.1	***If 'Abnormalities on F-DOPA' is equal to 'Yes' answer this question:***<br>Detected paragangliomas on F-DOPA	☐ Carotid body left<br>☐ Carotid body right<br>☐ Tympanic left<br>☐ Tympanic right<br>☐ Jugular left<br>☐ Jugular right<br>☐ Vagal left<br>☐ Vagal right<br>☐ Thoracic<br>☐ Abdominal<br>☐ Pheochromocytoma<br>☐ Other<br>☐ None<br>☐ Unknown
1.1.23.1.1	***If 'Detected paragangliomas on F-DOPA' is equal to 'Thoracic' answer this question:***	☐ Pulmonary

	Specification of thoracic paraganglioma	☐ Mediastinal<br>☐ Diaphragmatic<br>☐ Spine<br>☐ Para aortal<br>☐ Cardiac<br>☐ Aorticopulmonary<br>☐ Para tracheal<br>☐ Other
1.1.23.1.2	***If 'Detected paragangliomas on F-DOPA' is equal to 'Abdominal' answer this question:***<br>Specification of abdominal paraganglioma	☐ Mesenteric<br>☐ Para aortal<br>☐ Organ of Zuckerkandl<br>☐ Duodenal/ ampulla of Vater<br>☐ Bladder<br>☐ Colorectal<br>☐ Retroperitoneal<br>☐ Gonadal<br>☐ Peri-renal<br>☐ Sympathetic trunc<br>☐ Other
1.1.23.1.3	***If 'Detected paragangliomas on F-DOPA' is equal to 'Pheochromocytoma' answer this question:***<br>Location of pheochromocytoma	☐ Left adrenal gland<br>☐ Right adrenal gland<br>☐ Other
1.1.23.1.4	***If 'Detected paragangliomas on F-DOPA' is equal to 'Other' answer this question:***<br>Location of other paraganglioma	
1.1.23.2	***If 'Abnormalities on F-DOPA' is equal to 'Yes' answer this question:***<br>Change compared to the previous F-DOPA	☐ Growth<br>☐ Additional tumor<br>☐ Metastasis/metastases<br>☐ Ingrow in surrounding structure(s)<br>☐ Other<br>☐ None
1.1.23.2.1	***If 'Change compared to the previous F-DOPA' is equal to 'Other' answer this question:***<br>Other changes	
1.1.24	***If 'Imaging techniques' is equal to '68Ga-DOTA scan' answer this question:***<br>Date of 68Ga-DOTA scan	*(dd-mm-yyyy)*
1.1.25	***If 'Imaging techniques' is equal to '68Ga-DOTA scan' answer this question:***<br>Specific DOTA scan type	○ DOTATOC<br>○ DOTATATE<br>○ DOTANOC<br>○ Unknown

1.1.26	***If 'Imaging techniques' is equal to '68Ga-DOTA scan' answer this question:***<br>Abnormalities on 68Ga-DOTA?	◯ Yes<br>◯ No
1.1.26.1	***If 'Abnormalities on 68Ga-DOTA?' is equal to 'Yes' answer this question:***<br>Detected paragangliomas on DOTA	☐ Carotid body left<br>☐ Carotid body right<br>☐ Tympanic left<br>☐ Tympanic right<br>☐ Jugular left<br>☐ Jugular right<br>☐ Vagal left<br>☐ Vagal right<br>☐ Thoracic<br>☐ Abdominal<br>☐ Pheochromocytoma<br>☐ Other<br>☐ None<br>☐ Unknown
1.1.26.1.1	***If 'Detected paragangliomas on DOTA' is equal to 'Thoracic' answer this question:***<br>Specification of thoracic paraganglioma	☐ Pulmonary<br>☐ Mediastinal<br>☐ Diaphragmatic<br>☐ Spine<br>☐ Para aortal<br>☐ Cardiac<br>☐ Aorticopulmonary<br>☐ Para tracheal<br>☐ Other
1.1.26.1.2	***If 'Detected paragangliomas on DOTA' is equal to 'Abdominal' answer this question:***<br>Specification of abdominal paraganglioma	☐ Mesenteric<br>☐ Para aortal<br>☐ Organ of Zuckerkandl<br>☐ Duodenal/ ampulla of Vater<br>☐ Bladder<br>☐ Colorectal<br>☐ Retroperitoneal<br>☐ Gonadal<br>☐ Peri-renal<br>☐ Sympathetic trunc<br>☐ Other
1.1.26.1.3	***If 'Detected paragangliomas on DOTA' is equal to 'Pheochromocytoma' answer this question:***<br>Location of pheochromocytoma	☐ Left adrenal gland<br>☐ Right adrenal gland<br>☐ Other
1.1.26.1.4	***If 'Detected paragangliomas on DOTA' is equal to 'Other' answer this question:***	____________

	Location of other paraganglioma	
1.1.26.2	***If 'Abnormalities on 68Ga-DOTA?' is equal to 'Yes' answer this question:***<br>Change compared to the previous DOTA	☐ Growth<br>☐ Additional tumor<br>☐ Metastasis/metastases<br>☐ Ingrow in surrounding structure(s)<br>☐ Other<br>☐ None
1.1.26.2.1	***If 'Change compared to the previous DOTA' is equal to 'Other' answer this question:***<br>Other changes	
1.1.27	***If 'Imaging techniques' is equal to 'Other' answer this question:***<br>Date of other scan	*(dd-mm-yyyy)*
1.1.28	***If 'Imaging techniques' is equal to 'Other' answer this question:***<br>Abnormalities on other scan?	○ Yes<br>○ No
1.1.29	***If 'Imaging techniques' is equal to 'Other' answer this question:***<br>Type of other scan	
1.1.28.1	***If 'Abnormalities on other scan?' is equal to 'Yes' answer this question:***<br>Result of other scan	

Repeating Data 'Radiotherapy'

Form Radiotherapy

Number	Question	Answers
1.1	Radiotherapy for which paraganglioma	☐ Carotid body left<br>☐ Carotid body right<br>☐ Tympanic left<br>☐ Tympanic right<br>☐ Jugular left<br>☐ Jugular right<br>☐ Vagal left<br>☐ Vagal right<br>☐ Thoracic<br>☐ Abdominal<br>☐ Pheochromocytoma<br>☐ Other<br>☐ None<br>☐ Unknown
1.1.1	***If 'Radiotherapy for which paraganglioma' is equal to 'Thoracic' answer this question:***<br>Location of thoracic paraganglioma	☐ Pulmonary<br>☐ Mediastinal<br>☐ Diaphragmatic<br>☐ Spine<br>☐ Para aortal<br>☐ Cardiac<br>☐ Aorticopulmonary<br>☐ Para tracheal<br>☐ Other
1.1.1.1	***If 'Location of thoracic paraganglioma' is equal to 'Other' answer this question:***<br>Location of other thoracic paraganglioma	
1.1.2	***If 'Radiotherapy for which paraganglioma' is equal to 'Abdominal' answer this question:***<br>Location of abdominal paraganglioma	☐ Mesenteric<br>☐ Para aortal<br>☐ Organ of Zuckerkandl<br>☐ Duodenal/ ampulla of Vater<br>☐ Bladder<br>☐ Colorectal<br>☐ Retroperitoneal<br>☐ Gonadal<br>☐ Peri-renal<br>☐ Sympathetic trunc

		☐ Other
1.1.3	***If 'Radiotherapy for which paraganglioma' is equal to 'Abdominal' answer this question:***<br>Location of other abdominal paraganglioma	[ ]
1.1.4	***If 'Radiotherapy for which paraganglioma' is equal to 'Pheochromocytoma' answer this question:***<br>Location of pheochromocytoma	☐ Left adrenal gland<br>☐ Right adrenal gland<br>☐ Other
1.1.5	***If 'Radiotherapy for which paraganglioma' is equal to 'Other' answer this question:***<br>Other paraganglioma	[ ]
1.2	Indication of radiotherapy	○ Tumor growth<br>○ Hormone production<br>○ Tumor mutation<br>○ Pressure on surounding<br>○ Malignancy<br>○ Inoperable location<br>○ Patient's wish<br>○ Unknown<br>○ Other
1.3	Radiotherapy from the outside or inside?	○ Outside<br>○ Inside<br>○ Unknown
1.3.1	***If 'Radiotherapy from the outside or inside?' is equal to 'Outside' answer this question:***<br>Radiotherapy dosage	[ ] Gy
1.3.2	***If 'Radiotherapy from the outside or inside?' is equal to 'Outside' answer this question:***<br>Amount of radiotherapy fractions	[ ]
1.3.3	***If 'Radiotherapy from the outside or inside?' is equal to 'Inside' answer this question:***<br>Type of PRRT	○ 177-Lutetium<br>○ 68-Gallium<br>○ Other
1.3.3.1	***If 'Type of PRRT' is equal to 'Other' answer this question:***<br>Other PRRT	[ ]
1.3.4	***If 'Radiotherapy from the outside or inside?' is equal to 'Inside' answer this question:***<br>Amount of PRRT treatments	○ 1<br>○ 2<br>○ 3<br>○ 4<br>○ 5<br>○ 6<br>○ 7

		○ 8<br>○ 9<br>○ 10<br>○ >10
1.4	Complications due to radiotherapy	○ Yes<br>○ No<br>○ Unknown
1.4.1	***If 'Complications due to radiotherapy' is equal to 'Yes' answer this question:***<br>Experienced complication(s)	☐ Fatigue<br>☐ Hair loss<br>☐ Skin changes / fibrosis<br>☐ Swelling (edema)<br>☐ Tenderness<br>☐ Throat problems (e.g. trouble swallowing)<br>☐ Cough<br>☐ Shortness of breath<br>☐ Taste changes<br>☐ Hypothyreodism<br>☐ Diarrhea<br>☐ Nausea/vomiting<br>☐ Sexual problems<br>☐ Fertility problems<br>☐ Urinary and bladder problems<br>☐ Headache<br>☐ Blurry vision<br>☐ Long term: development of second cancer<br>☐ Lymphedema<br>☐ Mouth and gum sores<br>☐ Loss of appetite<br>☐ Other
1.4.1.1	***If 'Experienced complication(s)' is equal to 'Other' answer this question:***<br>Other experienced complication(s)	

Repeating Data 'Change in course of disease'

Form Change in course of disease

Number	Question	Answers
1.1	Date of follow up appointment *Warning shown if field's value is larger than NOW: 'Date cannot be in the future'*	(dd-mm-yyyy)
1.2	Symptoms	☐ Swelling in the neck<br>☐ Hoarseness<br>☐ Pain<br>☐ Difficulty swallowing<br>☐ Tinnitus<br>☐ Hearing loss<br>☐ Palpitations<br>☐ Excessive sweating<br>☐ Incidentaloma<br>☐ Dizziness<br>☐ Visual complaints<br>☐ Coughing<br>☐ Malignant hypertension<br>☐ Agitated<br>☐ No complaints<br>☐ Other<br>☐ Unknown
1.2.1	***If 'Symptoms' is equal to 'Other' answer this question:***<br>What other symptoms?	
1.3	Hormone production	○ Yes<br>○ No<br>○ Unknown
1.4	Tumor recurrence?	☐ Yes<br>☐ No
1.4.1	***If 'Tumor recurrence?' is equal to 'Yes' answer this question:***<br>Recurrence of which paraganglioma?	☐ Carotid body left<br>☐ Carotid body right<br>☐ Tympanic left<br>☐ Tympanic right<br>☐ Jugular left<br>☐ Jugular right

		☐ Vagal left<br>☐ Vagal right<br>☐ Thoracic<br>☐ Abdominal<br>☐ Pheochromocytoma<br>☐ Other<br>☐ None<br>☐ Unknown
1.4.1.1	***If 'Recurrence of which paraganglioma?' is equal to 'Other' answer this question:***<br>What other paraganglioma?	
1.5	Malignant tumor transformation?	○ Yes<br>○ No
1.5.1	***If 'Malignant tumor transformation?' is equal to 'Yes' answer this question:***<br>Malignant transformation of which PGL?	☐ Carotid body left<br>☐ Carotid body right<br>☐ Tympanic left<br>☐ Tympanic right<br>☐ Jugular left<br>☐ Jugular right<br>☐ Vagal left<br>☐ Vagal right<br>☐ Thoracic<br>☐ Abdominal<br>☐ Pheochromocytoma<br>☐ Other<br>☐ None<br>☐ Unknown
1.5.1.1	***If 'Malignant transformation of which PGL?' is equal to 'Thoracic' answer this question:***<br>Specification of thoracic paraganglioma	☐ Pulmonary<br>☐ Mediastinal<br>☐ Diaphragmatic<br>☐ Spine<br>☐ Para aortal<br>☐ Cardiac<br>☐ Aorticopulmonary<br>☐ Para tracheal<br>☐ Other
1.5.1.1.1	***If 'Specification of thoracic paraganglioma' is equal to 'Other' answer this question:***<br>Specification of other thoracic paraganglioma	
1.5.1.2	***If 'Malignant transformation of which PGL?' is equal to 'Abdominal' answer this question:***<br>Specification of abdominal paraganglioma	☐ Mesenteric<br>☐ Para aortal<br>☐ Organ of Zuckerkandl

		☐ Duodenal/ ampulla of Vater<br>☐ Bladder<br>☐ Colorectal<br>☐ Retroperitoneal<br>☐ Gonadal<br>☐ Peri-renal<br>☐ Sympathetic trunc<br>☐ Other
1.5.1.2.1	***If 'Specification of abdominal paraganglioma' is equal to 'Other' answer this question:***<br>Specification of other abdominal paraganglioma	
1.5.1.3	***If 'Malignant transformation of which PGL?' is equal to 'Pheochromocytoma' answer this question:***<br>Specification of malignant pheochromocytoma	☐ Left adrenal gland<br>☐ Right adrenal gland<br>☐ Other
1.5.1.3.1	***If 'Specification of malignant pheochromocytoma' is equal to 'Other' answer this question:***<br>Specification of other pheochromocytoma	
1.5.1.4	***If 'Malignant transformation of which PGL?' is equal to 'Other' answer this question:***<br>What other PGL has become malignant?	
1.6	Additional paraganglioma	○ Yes<br>○ No
1.6.1	***If 'Additional paraganglioma' is equal to 'Yes' answer this question:***<br>Location of additional paraganlioma(s)	☐ Carotid body left<br>☐ Carotid body right<br>☐ Tympanic left<br>☐ Tympanic right<br>☐ Jugular left<br>☐ Jugular right<br>☐ Vagal left<br>☐ Vagal right<br>☐ Thoracic<br>☐ Abdominal<br>☐ Pheochromocytoma<br>☐ Other<br>☐ None<br>☐ Unknown
1.6.1.1	***If 'Location of additional paraganlioma(s)' is equal to 'Thoracic' answer this question:***<br>Specification of thoracic paraganglioma	☐ Pulmonary<br>☐ Mediastinal<br>☐ Diaphragmatic<br>☐ Spine<br>☐ Para aortal<br>☐ Cardiac

		☐ Aorticopulmonary<br>☐ Para tracheal<br>☐ Other
1.6.1.1.1	***If 'Specification of thoracic paraganglioma' is equal to 'Other' answer this question:***<br>Specification of other thoracic paraganglioma	
1.6.1.2	***If 'Location of additional paraganlioma(s)' is equal to 'Abdominal' answer this question:***<br>Specification of abdominal paraganglioma	☐ Mesenteric<br>☐ Para aortal<br>☐ Organ of Zuckerkandl<br>☐ Duodenal/ ampulla of Vater<br>☐ Bladder<br>☐ Colorectal<br>☐ Retroperitoneal<br>☐ Gonadal<br>☐ Peri-renal<br>☐ Sympathetic trunc<br>☐ Other
1.6.1.2.1	***If 'Specification of abdominal paraganglioma' is equal to 'Other' answer this question:***<br>Specification of other abdominal paraganglioma	
1.6.1.3	***If 'Location of additional paraganlioma(s)' is equal to 'Pheochromocytoma' answer this question:***<br>Specification of pheochromocytoma	☐ Left adrenal gland<br>☐ Right adrenal gland<br>☐ Other
1.6.1.3.1	***If 'Specification of pheochromocytoma' is equal to 'Other' answer this question:***<br>Specification of other pheochromocytoma	
1.6.1.4	***If 'Location of additional paraganlioma(s)' is equal to 'Other' answer this question:***<br>Location of other paraganglioma	

Repeating Data 'Surgical interventions'

Form Surgical intervention

Number	Question	Answers
1.1	Date of surgical intervention *Exclude patient if field's value is larger than NOW with message: 'Date cannot be in the future.'*	___ ___ ___ *(dd-mm-yyyy)*
1.2	Type of excision	◯ Primary surgery<br>◯ Tumor recurrence<br>◯ Previous incomplete excision<br>◯ Other
1.2.1	***If 'Type of excision' is equal to 'Other' answer this question:***<br>Reason of other	
1.3	Which paraganglioma	◯ Carotid body left<br>◯ Carotid body right<br>◯ Tympanic left<br>◯ Tympanic right<br>◯ Jugular left<br>◯ Jugular right<br>◯ Vagal left<br>◯ Vagal right<br>◯ Thoracic<br>◯ Abdominal<br>◯ Pheochromocytoma<br>◯ Other<br>◯ None<br>◯ Unknown
1.3.1	***If 'Which paraganglioma' is equal to 'Thoracic' answer this question:***<br>Location of thoracic paraganglioma	☐ Pulmonary<br>☐ Mediastinal<br>☐ Diaphragmatic<br>☐ Spine<br>☐ Para aortal<br>☐ Cardiac<br>☐ Aorticopulmonary<br>☐ Para tracheal<br>☐ Other

1.3.1.1	***If 'Location of thoracic paraganglioma' is equal to 'Para tracheal' answer this question:***<br>Location of other thoracic paraganglioma	
1.3.2	***If 'Which paraganglioma' is equal to 'Abdominal' answer this question:***<br>Location of abdominal paraganglioma	☐ Mesenteric<br>☐ Para aortal<br>☐ Organ of Zuckerkandl<br>☐ Duodenal/ ampulla of Vater<br>☐ Bladder<br>☐ Colorectal<br>☐ Retroperitoneal<br>☐ Gonadal<br>☐ Peri-renal<br>☐ Sympathetic trunc<br>☐ Other
1.3.2.1	***If 'Location of abdominal paraganglioma' is equal to 'Other' answer this question:***<br>Location of other abdominal paraganglioma	
1.3.3	***If 'Which paraganglioma' is equal to 'Pheochromocytoma' answer this question:***<br>Location of pheochromocytoma	☐ Left adrenal gland<br>☐ Right adrenal gland<br>☐ Other
1.3.4	***If 'Which paraganglioma' is equal to 'Other' answer this question:***<br>Location of other paraganglioma	
1.4	Operation indication	☐ Tumor growth<br>☐ Hormone production<br>☐ Tumor mutation<br>☐ Pressure on surounding structures<br>☐ Malignancy<br>☐ Pregnancy wish<br>☐ Pregnancy<br>☐ Patient's wish<br>☐ Unknown<br>☐ Other
1.4.1	***If 'Operation indication' is equal to 'Other' answer this question:***<br>What other reason?	
1.5	Preoperative medical preparation	☐ Yes<br>☐ No

		☐ Unknown
1.5.1	***If 'Preoperative medical preparation' is equal to 'Yes' answer this question:***<br>What medical treatment?	☐ Alpha blocker<br>☐ Beta blocker<br>☐ Calcium channel antagonist<br>☐ Diuretics<br>☐ Vasodilators<br>☐ ACE inhitibors<br>☐ Benzodiazepines<br>☐ Other
1.5.1.1	***If 'What medical treatment?' is equal to 'Other' answer this question:***<br>Other medical treatment	
1.5.2	***If 'Preoperative medical preparation' is equal to 'Yes' answer this question:***<br>Response to medication	○ Therapy resistant<br>○ Hypotension<br>○ Normotension<br>○ Other
1.5.2.1	***If 'Response to medication' is equal to 'Other' answer this question:***<br>Other response	
1.6	PA available?	○ Yes<br>○ No
1.6.1	***If 'PA available?' is equal to 'Yes' answer this question:***<br>PA number of specimen	
1.7	Postoperative complications	○ Yes<br>○ No<br>○ Unknown
1.7.1	***If 'Postoperative complications' is equal to 'Yes' answer this question:***<br>Specification of postoperative complications	☐ Death<br>☐ Stroke<br>☐ Permanent cranial nerve injury<br>☐ Temporary cranial nerve injury<br>☐ Neurologic deficit<br>☐ Infection<br>☐ Hemorrhage/hematoma<br>☐ Other
1.7.1.1	***If 'Specification of postoperative complications' is equal to 'Permanent cranial nerve injury' answer this question:***<br>Permanent damage of cranial nerves	☐ n. IX<br>☐ n. X<br>☐ n. XI

		☐ n. XII<br>☐ n. VII<br>☐ n. VIII
1.7.1.2	***If 'Specification of postoperative complications' is equal to 'Temporary cranial nerve injury' answer this question:***<br>Temporary damage of cranial nerves	☐ n. IX<br>☐ n. X<br>☐ n. XI<br>☐ n. XII<br>☐ n. VII<br>☐ n. VIII
1.7.1.3	***If 'Specification of postoperative complications' is equal to 'Neurologic deficit' answer this question:***<br>Neurological deficit	☐ Horner Syndrome<br>☐ First bite syndrome<br>☐ Central nerve deficit<br>☐ Peripheral nerve deficit<br>☐ Laryngeus recurrens<br>☐ Other
1.7.1.3.1	***If 'Neurological deficit' is equal to 'Other' answer this question:***<br>Other neurological deficits	
1.7.1.4	***If 'Specification of postoperative complications' is equal to 'Other' answer this question:***<br>Other postoperative complication(s)	
1.8	Outcome of surgery	○ Radical<br>○ Irradical<br>○ Unknown
