# Supplementary material for: Head and Neck Paraganglioma (HNPGL) Registry: A study protocol for prospective data collection in patients with Head and Neck Paragangliomas
Source: PLoS One. 2024 Jul 25;19(7):e0307311. doi: 10.1371/journal.pone.0307311 (PMC11271953; doi:10.1371/journal.pone.0307311)
Supplement: S3 File — (PDF) [file pone.0307311.s003.pdf]

Head and Neck Paragangliomas Registry (nWMO)

Version 1.1 Feb 2023

PROTOCOL TITLE: Head and Neck Paragangliomas Multicenter Registry

**Short title (max 43 characters)**	**HNPGL multicenter registry**
**Version**	**8.0**
**Date**	**10-07-2032**
**Department**	**Vascular Surgery**
**Coordinating investigator/project leader**	Drs. J.M. de Bresser<br>Heidelberglaan 100<br>3584 CX Utrecht<br>The Netherlands<br>E-mail: j.m.debresser-4@umcutrecht.nl
**Principal investigator (in Dutch: hoofdonderzoeker/ uitvoerder)**	Dr. B.J. Petri, vascular surgeon<br>Heidelberglaan 100<br>3584 CX Utrecht<br>The Netherlands<br>E-mail: b.j.petri@umcutrecht.nl
**Other investigator(s)**	Dr. J.A. Rijken, head and neck surgeon, UMCU<br>Drs. M.J.C. van Treijen, endocrinologist, UMCU<br>Dr. B.P.M. van Nesselrooij, clinical genetics, UMCU<br>Prof. Dr. G.J. de Borst, vascular surgeon, UMCU

Sponsor (in Dutch: verrichter/opdrachtgever)	*UMC Utrecht*
**Subsidising party**	***N/A***
**Laboratory sites**	***N/A***

1. INTRODUCTION AND RATIONALE

Paragangliomas are rare neoplasms arising from the embryonic neural crest, stretching from the skull base to the pelvis. They are divided into two groups: PGLs associated with the parasympathetic system and PGLs linked to the sympathetic system. These highly vascularised tumours are benign in majority of the cases. In literature, less than 10 % is stated as malignant. [1, 2] Approximately 90 % of all PGLs occur in the adrenal paraganglia, hence the name pheochromocytoma. The remaining 10 % are extra adrenal tumours. This group is divided over the abdomen (85 %), thorax (12%) and the head and neck region (3 %). Most common localisations of these head and neck paragangliomas (HNPGL) are the carotid body (57%) [3], followed by the jugular foramen and vagal nerve.[4]

Symptoms associated with HNPGLs are a painless enlargement in the neck and signs associated with increasing growth of the tumour. Symptoms occur when the tumour presses on blood vessels, nerves and other structures in the surrounding area. In 20 % of the cases, PGLs secrete excess catechol amines. These hormones cause sympathetic symptoms like untreatable hypertension, headache, palpitations and excessive heavy sweating. [5, 6] Secreting tumours can be potentially lethal as symptoms can result in a hypertensive crisis.

Clinical incidence varies between 1/1000 000 and 1/ 100 000. Assumed is that these numbers may represent an underestimation because of the asymptomatic and clinically favourable nature of PGLs. [7, 8]

Therapy differs among the different types of tumours and there different subclasses. Wait-and-scan is for small tumours the golden standard of therapy since treatment can be more harmful than the disease.[9] Some tumour characteristics prefer a direct surgery approach over wait-and-scan. Indicators for direct surgery are some genetic mutations, e.g. *SDHB*-mutations[10], and production of catechol amines by the tumour. Tumour growth could be an indication to operate as growth causes compression of surrounding structures and thereby symptoms. Some tumours are too large for surgical excision. Therefore, additional techniques are introduced. They are favoured over, or in addition to radical surgery. Already existing techniques such as embolization, stenting and radiotherapy are implemented in options of care for patients. Only embolization or radiotherapy show a mild state of disease stabilization compared to complete surgical removal, but no RCTs are performed comparing data.[11] Surgery related complicates vary between major complications like death, stroke and permanent cranial nerve injury (PCI) and minor complications such as neurologic deficits – central as well as peripheral - , temporary cranial nerve injury (TCI), intra-or post-operative blood loss, wound infection and hematoma formation.

Genetic testing of tumours can reveal a possible underlying mutation. Different mutations cause different growth behaviour as well as different metastatic rates, thereby complicating the prediction of its growth behaviour. More understanding of underlying molecular mechanisms is needed to better predict growth.[12] The metastatic rate of PGL is estimated up to 4 – 6 %, but it is unclear which tumour will metastasize and which will not.[13]

Considering the serious adverse effects from intervention and the rarity of the tumour, these patients would greatly benefit from high quality studies and randomised controlled trials (RCT). However, RCTs will take years to produce results and are very costly. Therefore the aim of this registry is to build a large database that can be used for retrospective research. This registry can provide high quality long term follow-up data and will lead to the best care for patients with this rare disease. This cohort will greatly contribute in further research in the behaviour and familiar characteristics of these tumours.

2. OBJECTIVES

Primary Objective: to create a multicenter registry in order to collect specific data that can be used for future research on optimising diagnostic protocols, treatment strategies, improving symptom-free survival and optimising patient follow-up among HNPGL patients and patients with a genetic mutation associated with the development of head and neck paragangliomas.

3. REGISTRY DESIGN

This is a multicenter prospectively maintained observational cohort registry. The duration is for an indefinite amount of time. A list of participating centers can be found in Appendix 1.

4. REGISTRY POPULATION

4.1 Population (base)

Patients (aged 16 years and older, upon diagnosis), who are diagnosed with a genetic mutation associated with the development of HNPGL and/or one or more HNPGLs and are currently being treated or have been treated at the UMCU and other participating centers in the Netherlands, are eligible for this registry.

4.2 Inclusion criteria

In order to be included in the registry, a subject must meet all of the following criteria:

- A positive diagnosis for HNPGL or genetic mutation associated with the development of HNPGLs.
- Aged 16 years or older;

- Signed informed consent from patients.

4.3 Exclusion criteria

A potential subject who meets any of the following criteria will be excluded from participation in this registry:

- Individuals who are unable or unwilling to sign complete informed consent.

5. METHODS

5.1 Parameters/endpoints

5.1.1 Main parameter/endpoint

The main parameters of interest include, but are not limited to:

- Patient and treatment characteristics
- Patient diagnostics
- Symptom-free survival
- Treatment related complications
- Questionnaires related to the quality of life. Sent at the time of inclusion, after surgery and then after 1, 2, 5 years and then every 5 years via Castor.

The full list of parameters is presented in *Appendix 2.1, 2.2 and 2.3*. Depending on new insights on HNPGL these endpoints may be altered and updated.

5.2 Registry procedures

A patient information form (PIF) will be provided to the participant as well as verbal information by the treating physician (otolaryngology, vascular surgery, endocrinology or clinical genetics). As head and neck paraganglioma are very rare tumours, explanation of the study and additional questions by the patient can only be answered by the treating physician. If there are any questions which cannot be answered by the treating physician, the principal investigator can answer them. When the principal investigator is not available at that moment for the patient's questions, he will get in touch with the patient by phone or e-mail on a later moment. In addition, a written, dated, and signed broad consent form will be obtained by the participant and the treating physician/local principal investigator. In participating centers the local policy will be leading.

After obtaining broad consent, each participant is assigned a unique study ID. Consequently, all relevant clinical information will be extracted from the electronic health records (EHRs) and entered in the register's database in Castor EDC by members of the research team. Local participating centers will enter their own patients in their own secured Castor environment, specifically for their center. This is further specified in section 7.1. The above mentioned questionnaires (see 5.1.1) will be automatically sent via Castor by e-mail at set time points. It will take the participant approximately 15 - 20 minutes to complete the self-

administered questionnaires. In case of no response, patients will receive a reminder after 7 days to complete the questionnaire. The treatment, diagnostic imaging and laboratory test that subjects will undergo are standard care and indicated by the treating physician in accordance with the latest guidelines and will not be influenced by participation in the registry.

Diagnostic procedures and treatment

As this is an observational registry, decisions regarding diagnostic imaging and treatment strategy will be left to the discretion of the treating physician.

Patient follow-up

Patients will receive the questionnaires via Castor at certain set time points (5.1.1). No additional follow-up appointments will be added to the normal care of these patients. Follow-up will be done as for normal out-patient care.

5.3 Withdrawal of individual subjects

Subjects can leave the registry at any time for any reason if they wish to do so without any consequences. They can either submit the withdrawal form that is included in the patient information or reply to the invitation (e-)mail that they no longer want to participate. This is described in the patient information.

5.3.1 Specific criteria for withdrawal (if applicable)

Participation is voluntarily, the patient decides whether to participate or not.

5.4 Replacement of individual subjects after withdrawal

As it concerns a growing group of participants, no participants will be replaced.

5.5 Follow-up of subjects withdrawn from treatment

There will be no follow-up of subjects that are withdrawn from the registry and it has no consequences for any further treatment.

6. ETHICAL CONSIDERATIONS

6.1 Regulation statement

The registry will be designed and implemented according to ‘gedragscode gezondheidsonderzoek’ and ‘toetsingscriteria eenvormige toetsing’ that consist of the laws: ‘WGBO (Wet op de Geneeskundige BehandelingsOvereenkomst)’ and AVG (Algemene Verordening Gegevensbescherming’.

6.2 Recruitment and consent

Recruitment for patients will be the same in all participating centers. Eligible HNPGL patients or patients with a genetic defect causative of HNPGLs, have been informed about the HNPGL registry by their treating (principal) physician. Treating physicians are part of different medical specialties (radiologists, clinical genetics, otolaryngology specialists, vascular surgeons, and internal endocrinology specialists), all with experience for these specific tumours.

Subsequently they are presented the patient information form (PIF) and broad consent (BC) form by their treating physician. At their next physical visit to the hospital, at least 24 hours later, the patient is presented the opportunity to ask any questions about the registry. Questions can be answered by the primary investigator or treating physician. As patients often visit all specialists from four mentioned specialities (surgery; otolaryngology; clinical genetics; endocrinology) upon diagnosis, the patient can also discuss questions about the study with these physicians and sign broad consent with them. If, at any moment, the treating physician or physician of one of the other specialities cannot answer the questions, the primary investigator will be contacted. When the primary investigator cannot be reached, the patient will be contacted via e-mail or phone by the primary investigator within considerable time. If the patient wishes to participate in the registry, informed consent is obtained by the local principal investigator (PI), their treating physician or one of the other previously mentioned specialists and the BC form is signed by the patient and one of these physicians. The participant is presented a copy of the IC form. The IC forms are stored at a secured location at the participating center. Recruitment and consent of the patient is recorded in the electronic health record.

The UMC Utrecht recently received the status ‘Expertise Centre for patients with head and neck paraganglioma’. Every year, the UMC Utrecht receives around 20 new patients with this diagnosis, showing the rarity of these tumours. As this research doesn’t have any funding and only a hand full of specialists of the four mentioned specialties have knowledge of these tumours, there may occur moments where the treating physician is the primary investigator.

7. ADMINISTRATIVE ASPECTS, PRIVACY INCIDENTS AND END OF REGISTRY REPORT

7.1 Handling and storage of data and documents

Project data will be handled with uttermost discretion and is only accessible to authorized personnel who require the data to fulfil their duties within the scope of the research project. On the eCRFs and other project specific documents, participants are only identified by a

unique participant number. The PI stores the participant identification list locally – per participating center – in a secure place. Only authorized personnel receive access to the eCRF, where tracking of all alterations, deletions and other changes are logged. All authorized personnel using the eCRF are requested to use a two-step login to prevent misuse.
All participating centers will enter the data via Castor. Castor EDC is a browser-based, metadata-driven EDC software solution and workflow methodology for building and managing online databases. The eCRF contains data items as specified in this research protocol. Modification of the eCRF will be made only if deemed necessary and in accordance with an amendment to the research protocol. Access to the eCRF is password protected and specific roles are assigned (e.g. study coordinator, investigator, etc.). There will be a personalized access per center. All necessary pseudonymized patient data will be recorded in Castor. Due to the rarity of this disease and collecting prospective data, the multidisciplinarity (involving broad spectrum of terminology and abbreviations) and 'free text fiels in the EDP', the Research Data Platform (RDP) will only be used partially for data extraction. Questionnaires will be send via Castor. It is the responsibility of the PI (responsible for the research site) to assure that all data in the course of the study will be entered completely and correctly in the respective database. Pseudonymized MRI scans will be shared with the UMCU through Research Imaging Architecture (RIA). Original medical records and original records of clinical findings, related to the participant's progress shall be kept on site in corresponding patient folders.

The key file - per participating center - containing the combination of the participants unique study ID and its personal information (name and date of birth) will be stored at a secured location at the local participating center's network drive, only the local principal investigator, coordinating researcher and datamanager have access for identification purpose.

Additional radiological scans will be pseudonymized using RIA.

When needed for future research, the research data will be extracted and stored in one of following file format(s): .sav (SPSS), .r (R Statistics), .rm5 (Revman) in a secure research folder specifically created for that study. The research analyst, data manager, researcher, physician, principal investigator(s) and future HNPGL student assistant who will aid the researcher with gathering data, will get access to an export of the research data (made by one of the authorized people from the registry) gathered in the registry, which is placed within the new specific study folder, where additional gathering of data can be performed by authorized personnel. To be able to reproduce the research findings and to help future users

to understand and reuse the data all changes made to the raw data and all steps taken in the analysis will be documented in syntaxes and by using new versions of the database. The original database will not be altered and is stored on the research network disc of the division. The research data will be archived on the research network disc of the division for 10 years after the registry has ended. More details can be found in the datamanagement plan: https://dmponline.dcc.ac.uk/plans/85128

In case of data transfer with research centers within and outside of the European Union, an equivalent level of privacy will be pursued. No personal data or any patient traceable information will be shared, and data transfer agreements between centers will be obtained and documented.

7.2 Ethical Committee

Any research that will be conducted with (parts of) the data from this registry will be checked by the research quality coordinator of the division. Data will only be used for future research after a Confirmation Quality Check.

7.3 Privacy incidents

The person that notices a privacy incident, needs to report this immediately to the Central Coordinator of Information security (Dutch: Centrale Coördinator Informatiebeveiliging) stating the following information: contact information of reporter, description of the incident (including an indication of the impact on privacy) and actions that have been taken to control damage. A privacy incident is a security breach that can lead to loss or unlawful processing of personal data.

7.4 End of registry report

The investigator will report the end of study date, and later, the final report date, in Vidatum. The goal of this registry is to create a multicenter database that is unique in both size and long term follow-up. Hence we cannot define an specific end of this registry.

7.5 Publications

The UMCU has set up a database in accordance to this protocol. A join registry contract with local participating centers has made available that through a committee of members of the several participating centers, a research proposal can be examined and accepted for research. After acceptance by the committee, data from all centers will be made available in order to answer the proposed research question. Conform the contract, the

author should inquire potential collaborators who have provided data whether a potential collaborator wishes to be included on the list of collaborators.

The data from this registry will be used for publication in peer-reviewed international scientific journals and will be presented at (inter-) national scientific meetings. The future publication will be part of a PhD thesis on Head and Neck Paragangliomas. The researchers are responsible for the completeness and accuracy of publications and will comply with the guidelines for ethical publication of results. Researchers are responsible for the completeness and accuracy of publications. Researchers will comply with the guidelines for ethical publication of results.

7.6 Amendments

Amendments are changes made to the research after a Conformation Quality Check has been received. Any change that may cause the investigation to fall within the scope of the WMO is submitted to the ethical committee after a quality check by the research quality coordinator of your division. Other changes must undergo further review by the research quality coordinator of your division.

8. REFERENCES

1. Bastounis E, Maltezos C, Pikoulis E, Leppaniemi AK, Klonaris C, Papalambros E. Surgical treatment of carotid body tumours. *Eur J Surg*. 1999;165(3):198-202. doi:10.1080/110241599750007045
2. Y. Ü, A. A, A. Ö, et al. Carotid body tumors (Paragangliomas). *Asian Cardiovasc Thorac Ann*. 2001;9(3):208-211. doi:10.1177/021849230100900311 LK
3. Plukker JT, Brongers EP, Vermey A, Krikke A, van den Dungen JJ. Outcome of surgical treatment for carotid body paraganglioma. *Br J Surg*. 2001;88(10):1382-1386. doi:10.1046/j.0007-1323.2001.01878.x
4. Ozay B, Kurc E, Orhan G, et al. Surgery of carotid body tumour: 14 cases in 7 years. *Acta Chir Belg*. 2008;108(1):107-111.
5. Singh D, Pinjala RK, Reddy RC, Satya Vani PVNL. Management for carotid body paragangliomas. *Interact Cardiovasc Thorac Surg*. 2006;5(6):692-695. doi:10.1510/icvts.2006.135772
6. van der Bogt KEA, Vrancken Peeters M-PFM, van Baalen JM, Hamming JF. Resection of carotid body tumors: results of an evolving surgical technique. *Ann Surg*. 2008;247(5):877-884. doi:10.1097/SLA.0b013e3181656cc0
7. Baysal BE et al. Hereditary paraganglioma targets diverse paraganglia. *J Med Genet.* 2002;39`;617-622.
8. Oosterwijk, JC. Et al. First experiences with genetic counselling based on predictive DNA diagnosis in hereditary glomus tumours (paragangliomas). *J Med Genet.* 1996;33:379-383
9. Erdogan BA, Bora F, Altin G, Paksoy M. Our experience with carotid body paragangliomas. *Prague Med Rep*. 2012;113(4):262-270. doi:10.14712/23362936.2015.9
10. Bekele A, Kassahun A, Kassa S. A two years prospective follow up and outcome of patients operated for carotid body tumors: experience from Ethiopia. *Ethiop Med J*. 2012;50(4):325-330.

11. Power AH, Bower TC, Kasperbauer J, et al. Impact of preoperative embolization on outcomes of carotid body tumor resections. *J Vasc Surg*. 2012;56(4):979-989. doi:10.1016/j.jvs.2012.03.037
12. Gwon JG, Kwon T-W, Kim H, Cho Y-P. Risk factors for stroke during surgery for carotid body tumors. *World J Surg*. 2011;35(9):2154-2158. doi:10.1007/s00268-011-1167-7
13. Naik SM, Shenoy AM, Nanjundappa, et al. Paragangliomas of the Carotid Body: Current Management Protocols and Review of Literature. *Indian J Surg Oncol*. 2013;4(3):305-312. doi:10.1007/s13193-013-0249-4 LK

9. Appendix

Appendix 1: List of participating centres

Amsterdam UMC
Meibergdreef 9
1105 AZ Amsterdam

Erasmus Medisch Centrum (EMC)
Dr. Molewaterplein 40
3015 GD Rotterdam

Leiden Universitair Medisch Centrum (LUMC)
Albinusdreef 2
2333 ZA Leiden

Radboudumc
Geert Grooteplein Zuid 10
6525 GA Nijmegen

Appendix 2.1: List of parameters included in the registry

- Study number
- Year of birth
- Sex
- Specifics about referral to UMCU
- Date start of symptoms
- Medical history
- Symptoms specified
- Physical exam
- Affected side
- Radiology reports
- Radiology scans
- Genetic tumour mutation
- Pathogenicity of mutation
- Lab results
- Type of intervention
- Intervention details such as:
    - Complications
    - All relevant surgery reports
    - Anaesthesia reports
    - Duration of hospital stay
- Complications such as:
    - Death
    - Stroke
    - Permanent cranial nerve injury
    - Temporary cranial nerve injury
    - Neurologic deficits
    - Infections
    - Blood loss

    - Other specified complications
- Development of other paraganglioma beside HNPGL;
- Development of other tumours;
- Treatment of other tumours beside HNPGL
- Symptoms of enhanced metanephrines;
- Treatment of enhanced metanephrines
- Follow-up details such as:
    - Planned and unplanned hospital visits
    - Symptoms during follow-up
    - Physical exam during follow-up
    - Additional diagnostics during follow-up
    - Additional treatment during-follow-up
    - Reason lost to follow-up

Appendix 2.2: List of questionnaires

- European Organization for Research and Treatment for Cancer Quality of Life Questionnaire (EORTC QLQ-C30)
- EuroQuol 5D-5L (EQ-5D-5L)
- Hospital Anxiety and Depression Scale (HADS)
- Modified Fatigue Impact Scale (MFI-20)
- Short Questionnaire to Assess Health-enhancing Physical Activity (SQUASH)
- Work Ability Index (WAI)
- Cancer Worry Scale (CWS)

Appendix 2.3 List of possible genetic mutations causing head- and neck paragangliomas

- SDHA
- SDHB
- SDHC
- SDHD
- SDHAF2
- FH
- VHL
- EPAS1
- CSDE1
- MAML3
- RET
- NF1
- MAX
- TMEM127
- HRAS
